# The pea branching *RMS2* gene encodes the PsAFB4/5 auxin receptor and is involved in an auxin-strigolactone regulation loop

**DOI:** 10.1371/journal.pgen.1007089

**Published:** 2017-12-08

**Authors:** Yasmine Ligerot, Alexandre de Saint Germain, Tanya Waldie, Christelle Troadec, Sylvie Citerne, Nikita Kadakia, Jean-Paul Pillot, Michael Prigge, Grégoire Aubert, Abdelhafid Bendahmane, Ottoline Leyser, Mark Estelle, Frédéric Debellé, Catherine Rameau

**Affiliations:** 1 Institut Jean-Pierre Bourgin, INRA, AgroParisTech, CNRS, Université Paris-Saclay, Versailles, France; 2 Université Paris-Sud, Université Paris-Saclay, Orsay, France; 3 Sainsbury Laboratory Cambridge University, Bateman Street, Cambridge, United Kingdom; 4 Institute of Plant Sciences Paris-Saclay, INRA, CNRS, Université Paris-Sud, Université d'Evry, Université Paris-Diderot, Orsay, France; 5 Howard Hughes Medical Institute and Section of Cell and Developmental Biology, University of California San Diego, La Jolla, California, United States of America; 6 Agroécologie, AgroSup Dijon, INRA, Université Bourgogne Franche-Comté, Dijon, France; 7 LIPM, Université de Toulouse, INRA, CNRS, Castanet-Tolosan, France; Wake Forest University, UNITED STATES

## Abstract

Strigolactones (SLs) are well known for their role in repressing shoot branching. In pea, increased transcript levels of SL biosynthesis genes are observed in stems of highly branched SL deficient (*ramosus1* (*rms1*) and *rms5*) and SL response (*rms3* and *rms4*) mutants indicative of negative feedback control. In contrast, the highly branched *rms2* mutant has reduced transcript levels of SL biosynthesis genes. Grafting studies and hormone quantification led to a model where *RMS2* mediates a shoot-to-root feedback signal that regulates both SL biosynthesis gene transcript levels and xylem sap levels of cytokinin exported from roots. Here we cloned *RMS2* using synteny with *Medicago truncatula* and demonstrated that it encodes a putative auxin receptor of the AFB4/5 clade. Phenotypes similar to *rms2* were found in Arabidopsis *afb4/5* mutants, including increased shoot branching, low expression of SL biosynthesis genes and high auxin levels in stems. Moreover, *afb4/5* and *rms2* display a specific resistance to the herbicide picloram. Yeast-two-hybrid experiments supported the hypothesis that the RMS2 protein functions as an auxin receptor. SL root feeding using hydroponics repressed auxin levels in stems and down-regulated transcript levels of auxin biosynthesis genes within one hour. This auxin down-regulation was also observed in plants treated with the polar auxin transport inhibitor NPA. Together these data suggest a homeostatic feedback loop in which auxin up-regulates SL synthesis in an RMS2-dependent manner and SL down-regulates auxin synthesis in an RMS3 and RMS4-dependent manner.

## Author summary

Plant shoot branching results from the precise regulation of bud growth versus dormancy. Positive and negative feedback mechanisms are likely involved in the dynamic control of this highly plastic trait. Strigolactones, the most recently discovered class of plant hormones, play a key role in controlling shoot branching. Negative feedback control of strigolactone biosynthesis has been observed in several species and was shown in pea to be mediated by a shoot-to-root signal that is *RAMOSUS2* (*RMS2*)-dependent. The chemical nature of this feedback signal has been extensively discussed. Here, we demonstrate that the RMS2 protein belongs to the small family of auxin receptors and confirm that it behaves as an auxin receptor. Strigolactones decrease stem auxin levels by rapidly repressing transcript levels of auxin biosynthesis genes, thereby forming a long-distance feedback loop between auxin and strigolactones for the precise regulation of shoot branching in plants.

## Introduction

Feedback signals are an essential component of dynamic biological systems to enable robustness and plasticity in development. While negative feedback can attenuate signals, positive feedback can amplify or prolong them [1,2]. Several positive and negative feedback mechanisms are likely involved in the control of shoot branching, a sequential and life-long regulated process in plants. Shoot branching patterns are derived from axillary bud activation and branch growth. Axillary buds, located in the axils of most leaves, integrate a multitude of external and endogenous signals resulting in the decision to grow or remain dormant [3,4]. Negative feedback loops can limit excessive branching that may be detrimental to the plant and positive feedback loops can stimulate sustained bud outgrowth or maintain dormancy.

Strigolactones (SL) play a major role in regulating shoot branching and also act as rhizospheric signals [5–7]. Homeostasis of most plant hormones is achieved by feedback control of the biosynthetic pathway by the end-product, via the hormone signaling pathway [8–11]. Evidence for such negative feedback control of SL biosynthesis has been observed in several species, as highly branched SL-defective mutants possess increased transcript levels of SL biosynthesis genes and SL application can reduce these transcript levels [5,12–18]. In contrast with other hormones where this negative feedback is mediated by components of the hormone signaling pathway, for SL at least some of the feedback appears to be indirect [15].

The pea SL synthesis genes *RAMOSUS1* (*RMS1)* and *RMS5* encode two members of the CAROTENOID CLEAVAGE DIOXYGENASE family (PsCCD8 and PsCCD7 respectively, MORE AXILLARY GROWTH4 (MAX4) and MAX3 in Arabidopsis) [19,20]. These CCDs act downstream of the DWARF27 (D27) isomerase and together they catalyse the synthesis of carlactone, a key intermediate in SL biosynthesis [21]. Downstream of the two CCDs, different enzymes including the cytochrome P450 (MAX1) and LATERAL BRANCHING OXIDOREDUCTASE (LBO) are involved in the synthesis of bioactive SL or SL-like compounds [22–25]. Carlactone-derived compounds with a butenolide ring (D ring) connected to a tricyclic lactone (ABC rings) via an enol-ether bridge are defined as canonical SLs. The pea *RMS3* and *RMS4* genes, required for SL response, encode the SL receptor (AtD14 in Arabidopsis) and an F-box protein (MAX2 in Arabidopsis), respectively [20,26]. The SL receptor hydrolyses SL to form a complex with the D-ring product. This complex undergoes a conformational change and binds to the MAX2/RMS4 F-box protein, a subunit of an Skp-Cullin-F-box (SCF) E3 ubiquitin ligase complex [26,27]. In the SL signalling pathway, the ubiquitination and proteasome-mediated degradation targets of this D14/SCF^MAX2^ complex include the SL repressor proteins D53 in rice and SMXL6-SMXL8 in Arabidopsis [28–31]. These proteins can function as transcriptional repressors by recruiting the corepressors TOPLESS and TOPLESS-RELATED (TPR) [29–31], although SMXL7 retains significant function when the TPL interaction domain is deleted [32]. There is good evidence that one transcriptional target for the SMXLs in the control of shoot branching is inhibition of transcription of the TEOSINTE BRANCHED1, CYCLOIDEA, PCF (TCP) transcription factor family member *BRC1* [30,31]. Expression of *BRC1*, localized in axillary buds, is upregulated by SLs in some species [33,34]. In pea and Arabidopsis, the shoot branching and dwarf phenotypes of the *brc1* mutant are less pronounced than those of SL deficient (*max3/rms5*, *max4/rms1*) and SL response (*max2/rms4*, *Atd14/rms3*) mutants, suggesting other systemic functions for SL [33,35–37]. In Arabidopsis, SL can repress the main stem polar auxin transport stream (PATS) via rapid removal of the PIN FORMED1 (PIN1) auxin efflux protein from the basal plasma membrane of xylem parenchyma cells [38–42]. The SMXL6-SMXL8 proteins appear to be involved in the SL regulation of PIN1 accumulation at the plasma membrane by an unknown mechanism which is unlikely to be transcriptional [40].

In pea, grafting studies and hormone quantifications of highly branched *rms* mutants (*rms1* to *rms5*) led to a model for shoot branching control involving two novel, long-distance, graft-transmissible, signals [43–46]: a root-to-shoot branching inhibitor, now identified as SL [5,6] and an unknown shoot-to-root feedback signal dependent on *RMS2* [47]. This *RMS2*-dependent feedback signal was proposed to positively regulate SL synthesis gene transcript levels and to negatively regulate xylem-sap cytokinin (X-CK) export from roots, as SL synthesis and signalling mutants all possess greatly increased *RMS1* transcript levels and reduced X-CK levels, whereas *rms2* mutants have low levels of *RMS1* and *RMS5* transcripts and increased X-CK export [20,44]. The additive branching phenotype of *rms1 rms2* double mutants in comparison with single mutants supported this model where *RMS1* and *RMS2* controlled two different long-distance signals [48]. Based on grafting studies demonstrating movement of the *RMS2*-dependent feedback signal in a shoot-to-root direction, the feedback control of SL was proposed to be mostly indirect because SL can only move in a root-to-shoot direction [15,43,46,49]. Feedback regulation of SL biosynthesis gene transcripts was also found to occur in SL mutants of Arabidopsis [15], rice [17,18,50], petunia [16,51,52], maize [13] and the moss *Physcomitralla patens* [12]. Application of the synthetic SL, GR24, can down-regulate the transcript levels of SL biosynthesis genes [5,12,13]. The Arabidopsis *max1* to *max4* mutants also display a strong reduction in X-CKs, with reciprocal grafting experiments between WT and *max2* (*rms4*) indicating that X-CK exported from roots is mostly shoot-regulated, as shown in pea [49,53].

The chemical nature of the *RMS2*-dependent feedback signal has been extensively discussed [54,55]. In pea, two feedback signals were proposed in branching control: a branch-derived signal, very likely auxin, and the RMS2-dependent feedback signal [55]. Since the *rms2* mutant has high IAA levels and is able to respond to IAA, it was also suggested that the *RMS2*-dependent feedback signal was auxin-independent, although auxin and the feedback signal share similar characteristics [54]. In pea and Arabidopsis, treatments that decrease stem auxin levels (decapitation, IAA polar transport inhibitors, defoliation etc.) also reduce transcript levels of the SL biosynthesis genes in the same tissues [15,44]. Auxin application to the decapitated stump or to intact plants results in an increase in transcript abundance of *CCD7* (*RMS5/MAX3/HTD1*) and *CCD8* (*RMS1/MAX4/D10*) in pea [20,44], Arabidopsis [56], rice [17,57] and maize [13]. Auxin is also known to rapidly reduce CK biosynthesis [58]. In particular, decapitation rapidly increases the transcript levels of CK biosynthesis genes in pea stem nodes [59] and X-CK levels in bean [60], whereas IAA applied to the decapitated stump prevents these augmentations. In Arabidopsis, it was proposed that IAA up-regulates the SL biosynthesis gene, *CCD7* (*AtMAX3*) via the AXR1-dependent pathway in the basal inflorescence stem [15], and in the hypocotyl [56]. AXR1 functions in the activation of SCF complexes by rubinylation [61] and mutations in *AXR1* confer auxin resistance [62]. In the basal stem of Arabidopsis *axr1 max2* double mutants, *MAX3* transcript levels are considerably reduced in comparison to *max2*, but not completely restored to WT levels [15]. These results are similar to analyses of *RMS1* transcript levels in the epicotyl of *rms1 rms2* double mutants [48] and altogether strongly suggest the involvement of auxin in feedback regulation of SL biosynthesis gene expression.

Here we show that the *RMS2* gene encodes an F-box protein of the small family of auxin receptors including the TRANSPORT INHIBITOR RESPONSE1/AUXIN-SIGNALING F-BOX (TIR1/AFB) proteins, with RMS2 belonging to the AFB4/AFB5 clade. We demonstrate that transcript levels of IAA biosynthesis genes are rapidly down-regulated by SL application and propose a model whereby SL and IAA regulate each other’s metabolism, highlighting the importance of homeostatic systems in shoot branching control.

## Results

### The *RMS2* pea branching gene encodes the PsAFB4/5 auxin receptor

**Fig 1:**
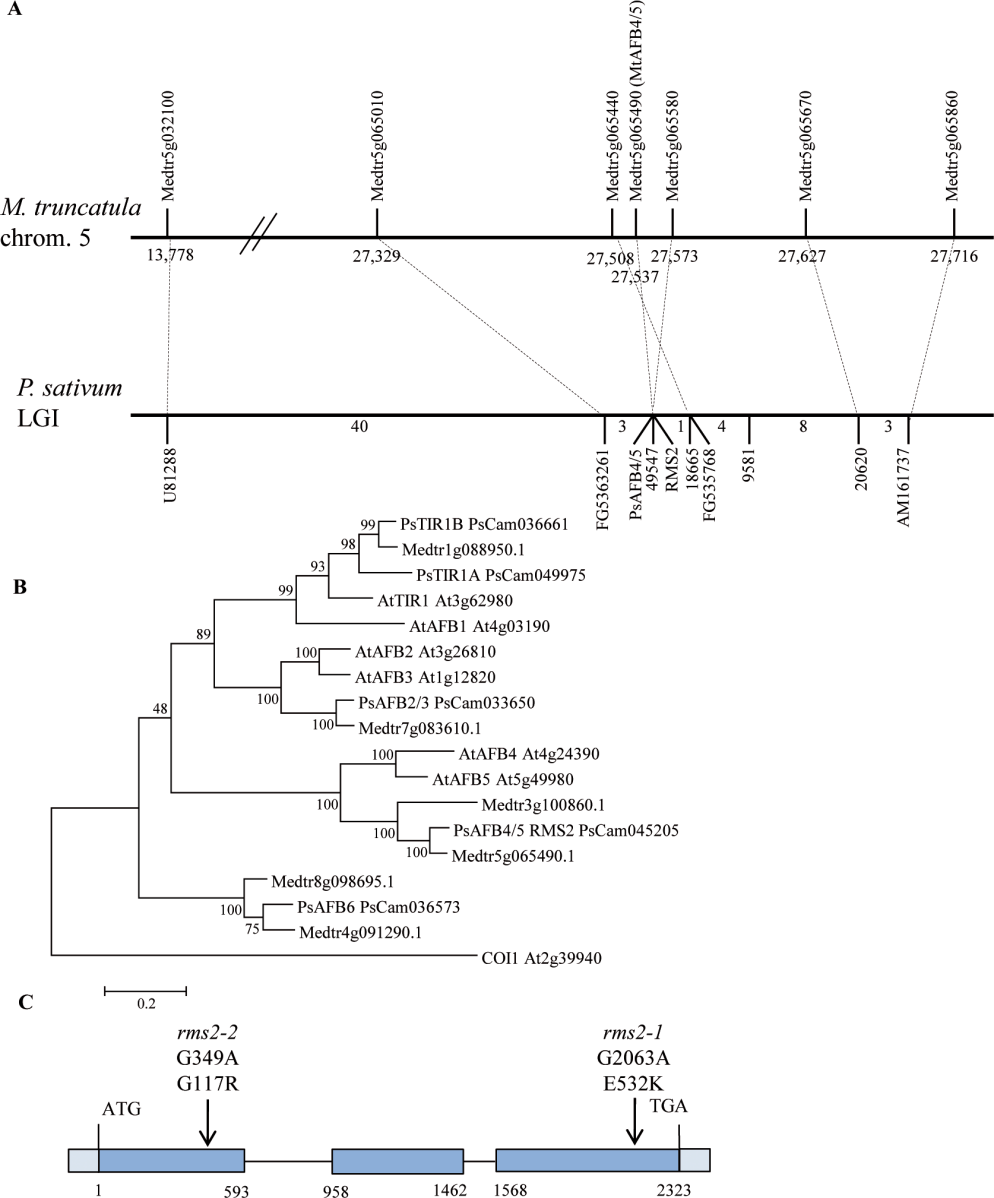
*RMS2* encodes the pea orthologue of AFB4/5 from Arabidopsis of the TIR1/AFB auxin receptor family. **(A)** *RMS2* locus positional cloning. The position of the *M*. *truncatula* chromosome 5 genes used to define markers in *P*. *sativum* (based on the conservation of synteny between the two species) are indicated in Mbp according to *M*. *truncatula* A17 genome assembly 4.0 (http://jcvi.org/medicago/). The number of recombinants between the adjacent markers (in an F2 population of 528 individuals) is indicated below the linkage group I pea genetic map. The dotted lines indicate putative orthologous relationships between pea and *Medicago* markers. **(B)** Phylogenic tree of the TIR1/AFB auxin receptor family from Arabidopsis, pea and *Medicago truncatula*. Protein sequences were aligned and used to generate the Maximum Likelihood phylogenetic tree with 1,000 bootstrap replicates. The percentage of replicate trees in which the associated taxa clustered together in the bootstrap test are shown next to the branches. Analyses were conducted in MEGA7 [111]. **(C)** Structure of the *RMS2* gene and positions of the *rms2* mutations. Amino acid substitutions resulting from single nucleotide substitutions are shown. Exons are depicted as blue boxes, introns as black lines.

The *RMS2* gene had been mapped previously to linkage group (LG) I of the pea genetic map in a large region containing the classical markers ENOD40, sym19 and PsU81288 [63–65]. These three markers were also found to be linked in *Medicago truncatula* (*Mt*) where chromosome 5 corresponds to pea LGI. We looked for candidate genes located in this region that were likely to play a role in hormone signaling, particularly auxin signaling, and plant architecture [54]. Taking advantage of the good conservation of synteny between *Mt* chromosome 5 and pea LGI, we identified pea genetic markers in the vicinity of these candidate genes and mapped them in an F2 pea mapping population of 528 individuals derived from a cross between K524 (*rms2*) and JI281 [66,67]. Three markers (FG5363261, AM161737, FG535768) corresponding to Medtr5g065010, Medtr5g065860, and Medtr5g065440, respectively, and located near Medtr5g065490, a putative auxin receptor of the TIR1/AFB family, were tightly linked to *rms2* in pea (Fig 1A). The sequence of the pea orthologue of Medtr5g065490, PsCam045205, and of other pea homologues of the TIR1/AFB family of auxin receptors were obtained from the pea RNA-Seq gene atlas (http://bios.dijon.inra.fr/FATAL/cgi/pscam.cgi); [68]. PsCam045205 has been mapped on LGI using different pea mapping populations [69]. Phylogenetic analysis indicated that PsCam045205 belongs to the Arabidopsis AFB4/AFB5 clade. The pea Unigene set described in [68] represents most of the expressed genes of pea and was derived from several cDNA libraries. Therefore it is very likely that PsCam045205 is the only pea AFB homologue in this clade (Fig 1B). PsAFB4/5 was sequenced in the two available *rms2* mutants and in their respective wild-type lines. Mutations were found for each *rms2* mutant. The *rms2-1* mutation (line K524 from Torsdag) leads to the replacement of glutamic acid by lysine at position 532 and the *rms2-2* mutation (line W5951 from Parvus) leads to the replacement of glycine by arginine at position 117 (Fig 1C and S1 Fig). Both mutations affect amino acids located close to those residues forming the IAA binding pocket of the TIR1 homologue (S1 Fig, [70]). Taken together, these data demonstrate that *RMS2* likely corresponds to *PsAFB4/5*.

An *in vitro* stem segment assay was used to investigate IAA responses in *rms2* as it was previously shown that transcript levels of the SL biosynthesis gene *RMS1* are increased in isolated stem segments treated with IAA [44]. Internode 4–5 of 16-d-old plants harvested from WT^Térèse^, *rms1-10*, *rms2-1* and *rms1-10 rms2-1* double mutant plants were treated with a 10 μM IAA solution for 3 h. Transcript levels of *RMS1* and *RMS5*, together with the pea homologue of the rice *D27* gene *PsD27* were analyzed. Phylogenetic analysis indicated that PsD27 is in the same clade as the rice D27 and the two proteins share 59% identity. Increased transcript levels of all three SL biosynthesis genes were observed in WT and *rms1-10* internodes treated with IAA compared to mock controls (S2 Fig). In contrast, the increase in *RMS1*, *RMS5* and *D27* transcript levels in response to IAA was either abolished or attenuated in mutants containing the *rms2-1* mutation. These results suggest that transcript levels of SL biosynthesis genes are stimulated by IAA and that this induction is impaired in plants containing the *rms2* mutation.

### Mutations in the pea *RMS2* gene and in the Arabidopsis *AFB4/AFB5* genes confer similar phenotypes

**Fig 2:**
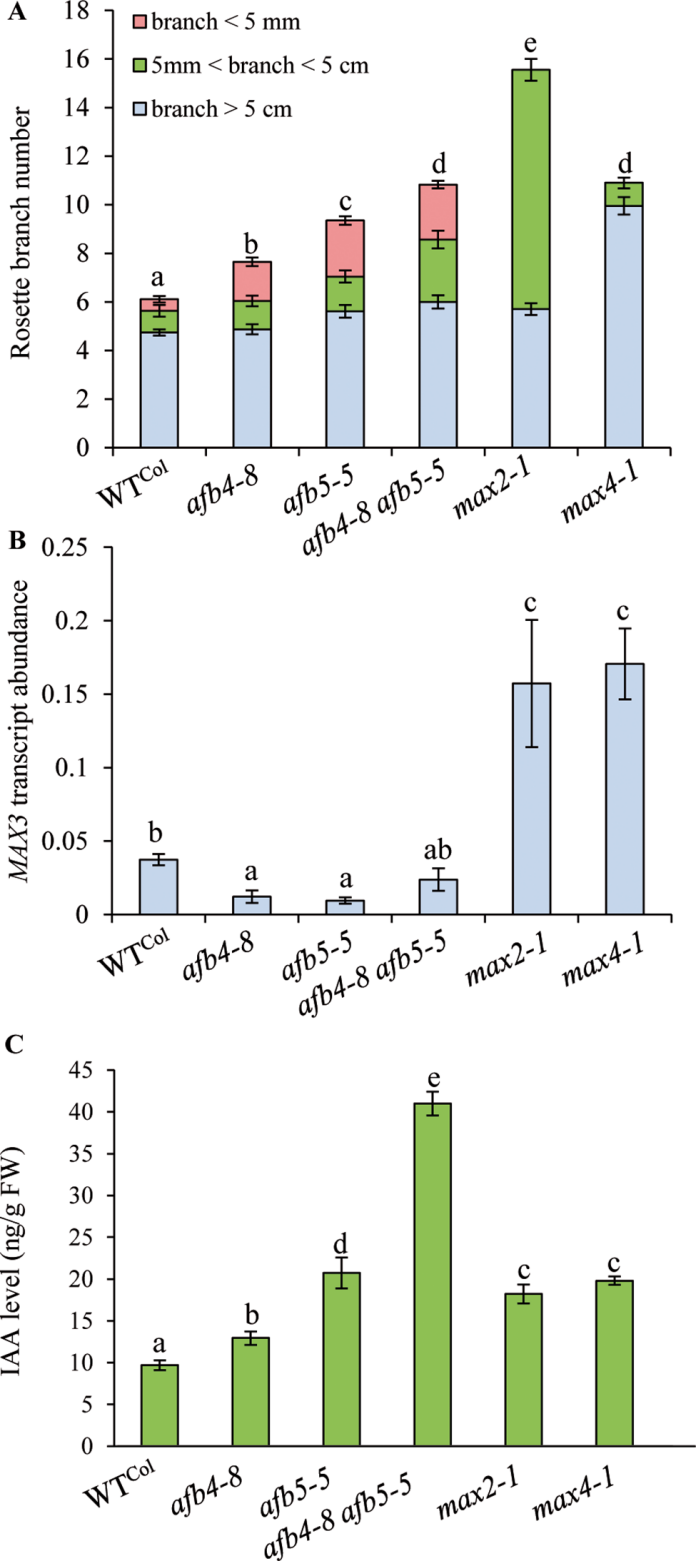
Arabidopsis mutants in the AFB4/5 clade have similar phenotypes as the pea *rms2* mutant. **(A)** The *afb4-8* and *afb5-5* mutations confer branching phenotypes. Total number of rosette branches was measured on 40 day-old plants and branches classified according to their length (n = 19–24). Different letters indicate significantly different results for the total number of rosette branches based on a Kruskal–Wallis test (P < 0.05). **(B)** Transcript abundance of *MAX3* in basal stems of Arabidopsis. *MAX3* transcript abundance relative to *ACTIN* in various genotypes of 40 day-old plants (n = 3 pools of 10–15 plants). Different letters indicate significantly different results based on a Kruskal–Wallis test (P < 0.05). **(C)** IAA (in ng per g fresh weight) in basal stems of 40 day-old Arabidopsis plants (n = 4 pools of 12–15 plants). Different letters indicate significantly different results based on a Kruskal–Wallis test (P < 0.05). For A-C bars represent means ± SE.

To investigate whether pea *RMS2* and Arabidopsis *AFB4/5* perform similar functions in shoot branching regulation, we analysed the branching phenotypes of single and double *Arabidopsis afb4* and *afb5* mutants, as well as *max* mutants, and examined SL biosynthesis gene transcript and auxin levels which are known to be altered in *rms2* [66]. The pea *rms2* mutants display increased shoot branching, particularly at basal nodes [71]. Single and double *afb* mutants had levels of rosette branching that were intermediate between WT and the highly branched *max2-1* mutant (Fig 2A). The *afb4-8 afb5-5* double mutant had a similar number of axillary branches as the SL deficient *max4-1* mutant. Interestingly, the classification of axillary branches into three groups according to their length showed that *afb4-8*, *afb5-5* and *afb4-8 afb5-5* mutants possess a larger proportion of small branches (< 5 mm) in comparison to WT and the *max* mutants. This particular branching phenotype is also found in the pea *rms2* mutants, which displays long basal branches and small branches at upper nodes, whereas SL mutants have long branches at most nodes [71].

In pea, *RMS2* was proposed to play a role in the feedback regulation of *RMS1* (*PsCCD8*) expression because *RMS1* transcript levels are greatly up-regulated in all *rms* mutants except for *rms2* [44]. To test if *afb* mutants have similarly low levels of SL biosynthesis genes, *MAX3* (*AtCCD7*) expression was quantified in adult basal stems of the *afb* and *max* mutants. *MAX3* transcript levels were increased in *max2-1* and *max4-1*, but were similar or lower than WT in the single and double *afb* mutants (Fig 2B). Thus, both *rms2* and *afb* mutants possess reduced SL biosynthetic gene transcript abundance. Another physiological trait of pea *rms2* mutants is the increased stem level (up to 5 fold higher than WT) of the predominant auxin indole-3-acetic acid (IAA) [66]. IAA levels were also found to be higher in *afb4-8* and *afb5-5* single mutants and up to 4 fold higher than WT in *afb4-8 afb5-5* double mutants (Fig 2C).

**Fig 3:**
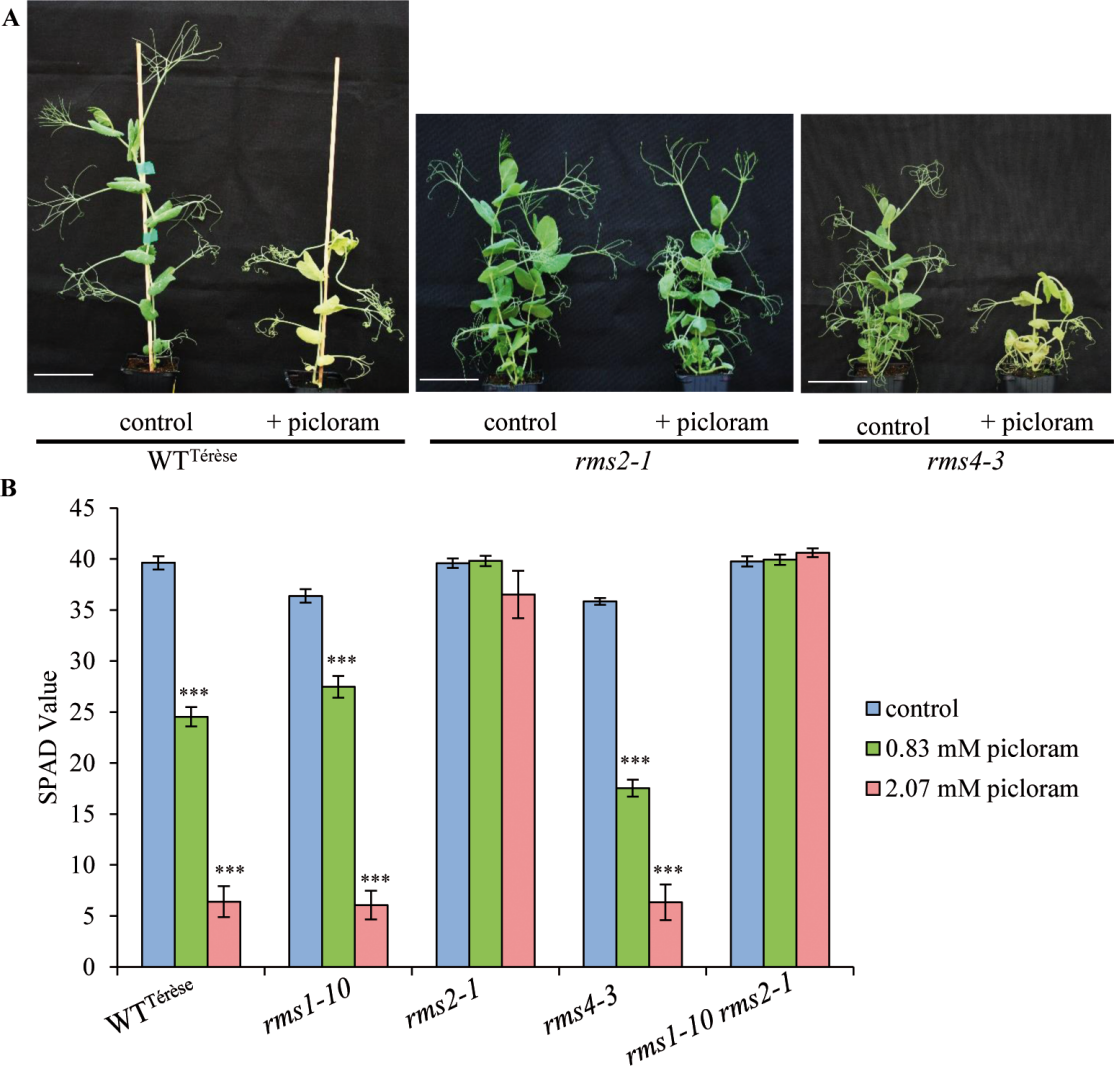
The *rms2-1* pea mutant is picloram resistant. **(A)** Two week-old plants were sprayed with ~3 ml 0 mM or 0.83 mM picloram solutions. Representative plants are shown 10 days after treatment. Scale bar = 6 cm. **(B)** SPAD values (determined with a Soil Plant Analysis Development chlorophyll meter) of two week-old plants sprayed with 0 mM, 0.83 mM or 2.07 mM picloram solutions after 8 days (n = 12). Asterisks denote significant differences between treated and corresponding control plants based on a post-hoc Kruskal–Wallis test (P < 0.001). Data represent means ± SE.

It was previously reported that the *Arabidopsis afb5* mutant, and to a lesser extent *afb4*, show specific resistance to the herbicidal auxin picloram (4-amino-3,5,6-trichloropicolinic acid) [72,73]. The resistance of the *rms2* pea mutants to this synthetic picolinate auxin was therefore investigated. A foliar spray of 0.83 mM picloram was applied to 20-d old plants of WT^Térèse^ (Térèse background), *rms2-1*, and *rms4-3* mutants. After 10 days, depigmentation was observed in all genotypes and severe auxin-related symptoms including stem curvature and foliar curling were observed in all genotypes except for *rms2-1*which exhibited limited foliar curling (Fig 3A). To quantify picloram resistance, the chlorophyll content was estimated with a Soil Plant Analysis Development (SPAD) chlorophyll meter in WT^Térèse^, *rms1-10*, *rms2-1*, *rms4-3* mutants and *rms1-10 rms2-1* double mutants 8 days after treatment with picloram (0.83 mM or 2.07 mM). A strong picloram dose-dependent decrease in chlorophyll content was observed for all genotypes except for *rms2-1* and *rms1-10 rms2-1* double mutants, which were resistant even at the higher dose (Fig 3B). The picloram resistance of the *rms2-1* (Torsdag background) and *rms2-2* (Parvus background) mutant alleles were also confirmed (S3 Fig). These results demonstrate that picloram resistance is conferred by the two pea *rms2-1* and *rms2-2* mutations, similar to that observed for the *Arabidopsis afb5-1* mutant, and to a lesser extent *afb4-8*.

### RMS2 protein can bind Aux/IAA proteins from *Arabidopsis* in an IAA-dependent way and can bind ASK1 constitutively

**Fig 4:**
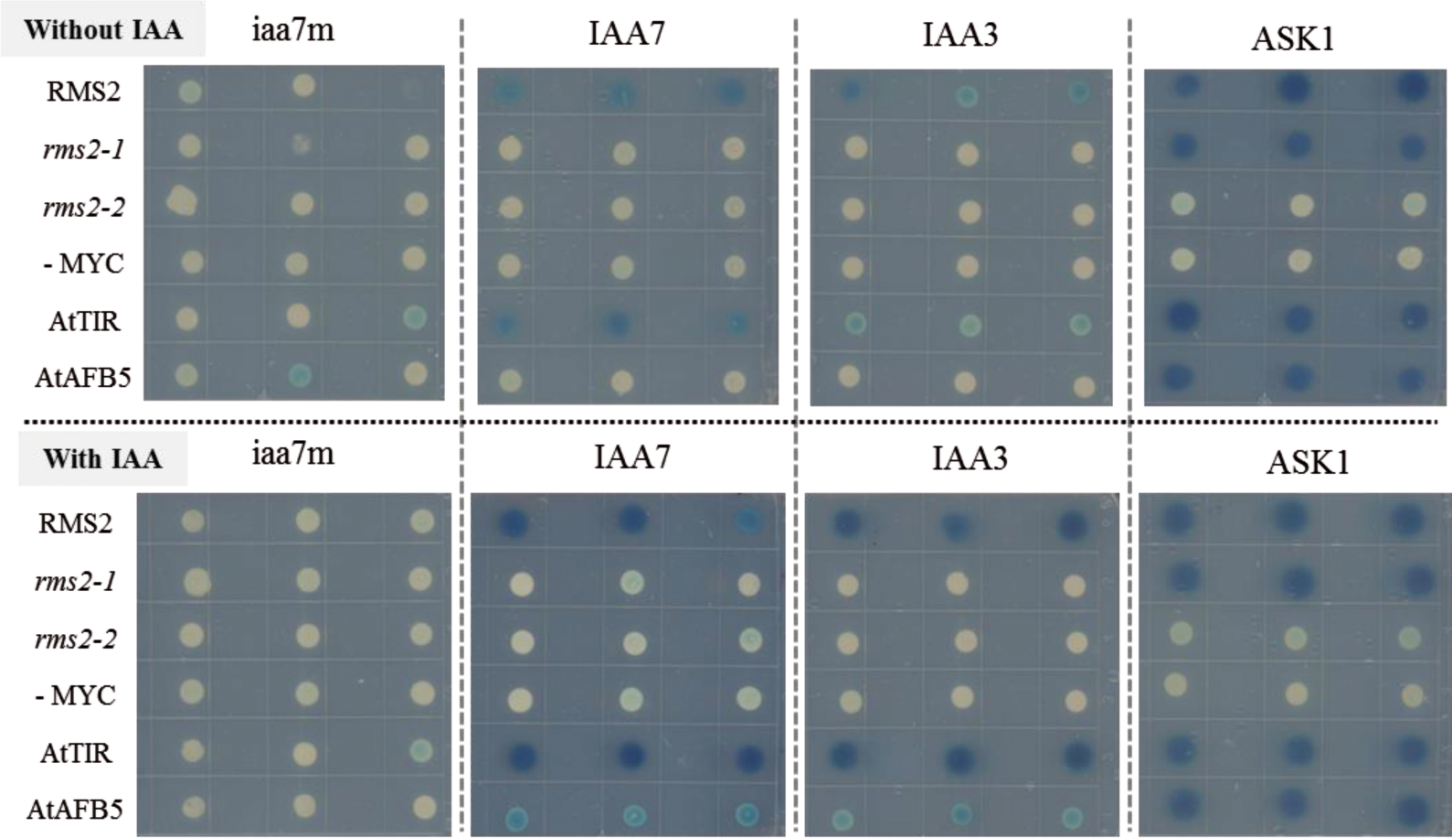
RMS2 protein can bind Aux/IAA proteins in the presence of IAA and to ASK1 protein IAA-independently. The lexA DNA-binding domain (lexA) was fused to RMS2 (WT), rms2-1, rms2-2 AtTIR1, AtAFB5, and the cMyc epitope while the B42 activation domain (AD) was fused to Arabidopsis iaa7m (with degron substitutions), IAA7, IAA3, and ASK1. Three independent transformants containing LexA–RMS2/TIR1/AFB5/Myc and B42–Aux/IAAs/ASK1 were spotted in selective media with and without 50 μM IAA. Blue product released by β-galactosidase reporter activity is a measure of protein-protein interactions.

Auxin perception and signaling by TIR1/AFBs require the binding of TIR1/AFBs to ASK1 (ARABIDOPSIS SKP1 HOMOLOG1), a core component of the SCF complex [74]. Interaction between the Arabidopsis ASK1 and pea RMS2 proteins (from WT, *rms2-1* and *rms2-2* mutants) was therefore tested in a yeast two-hybrid (Y2H) system. ASK1 was shown to interact with both WT RMS2 and mutant rms2-1 proteins, but not rms2-2, likely due to the location of the rms2-2 mutation near the F-box domain (Fig 4 and S1 Fig). Thus, RMS2 can interact with ASK1 and can presumably form an SCF complex. To investigate whether pea RMS2 can function as an auxin co-receptor, the Y2H system was used to test for interactions between pea RMS2 proteins (from WT, *rms2-1* and *rms2-2* mutants) and *Arabidopsis* IAA7 and IAA3 proteins in the presence or absence of IAA. We chose these two Aux/IAA proteins because IAA7 is known to interact with *Arabidopsis* AFB5 and other auxin receptors, whereas IAA3 does not interact with AFB5 [75]. TIR1 interactions were assessed as a positive control. Similar to TIR1, RMS2 interacted with IAA7, even when IAA was not present. The addition of IAA appeared to increase the binding of both TIR1 and RMS2 to IAA7. In our experiment, some interaction was observed in the absence of IAA between IAA3 and TIR1 or RMS2, but not AFB5. This interaction was strongly enhanced in the presence of IAA. For both rms2-1 and rms2-2 mutant proteins, no interaction with IAA7 or IAA3 was detected in either presence or absence of auxin (Fig 4 and S4 Fig). The iaa7m protein has three substitutions in the degron sequence and did not interact with any of the AFBs. These results indicate that pea RMS2 can bind Aux/IAA proteins in an IAA-dependent manner, though no specificity for the IAA3 or IAA7 co-receptor partner was observed for proteins in the AFB4/5 clade.

### Strigolactones repress IAA levels in pea stems via RMS3 and RMS4

**Fig 5:**
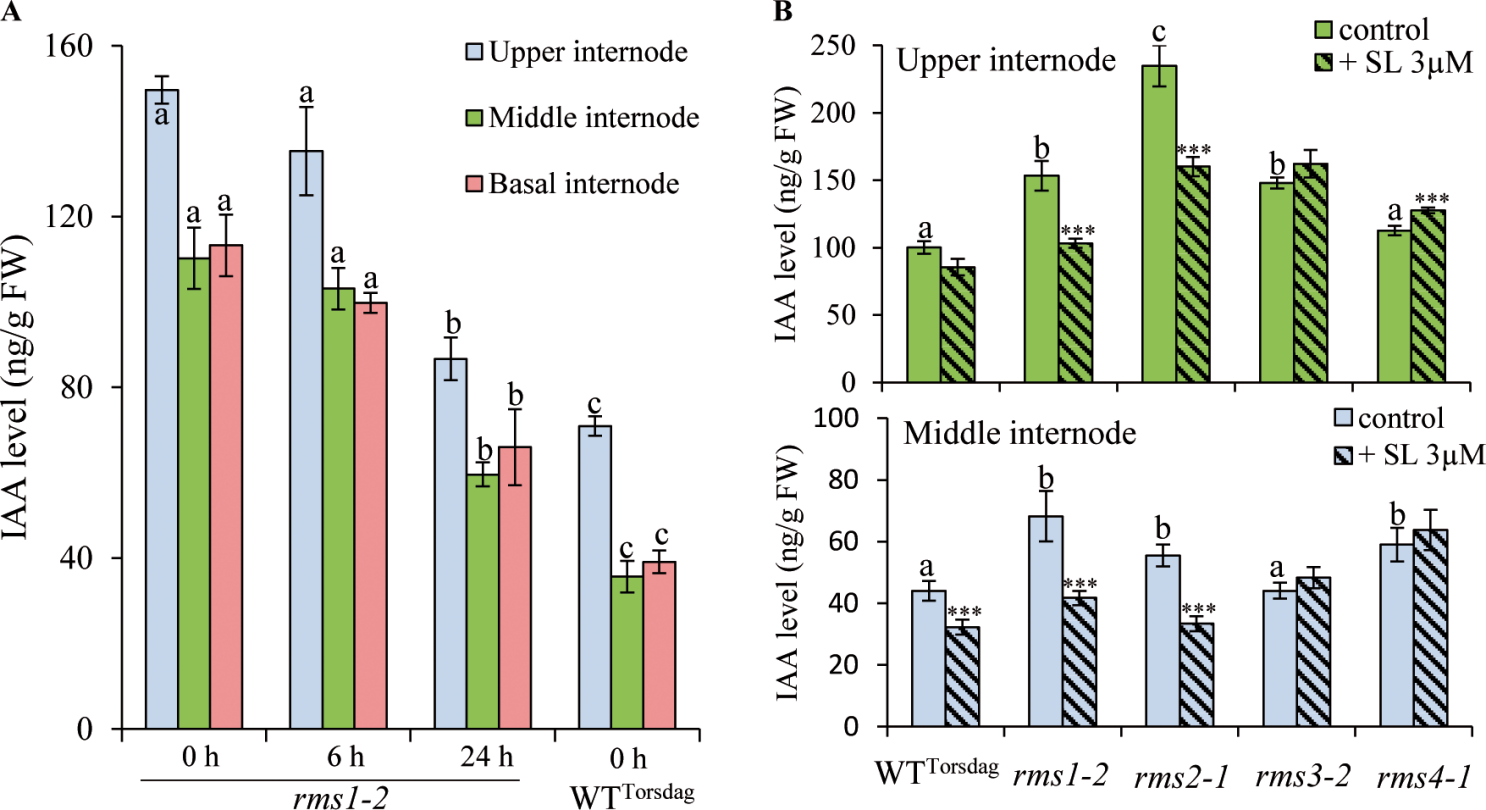
Strigolactone application down-regulates IAA level in pea stems via *RMS3* and *RMS4*. **(A)** IAA level (in ng per g fresh weight) was measured in 3 week old WT (Torsdag) and *rms1-2* plants treated hydroponically with 3 μM (±)-3'-Me-GR24 for 6 or 24 h or with control solution (0 h); WT was treated only with control solution. Upper internodes below the apex (internode 9–10), middle internodes (internode 6–7) and basal internodes (internode 3–4) were analysed (n = 4 pools of 8–10 plants each). Different letters indicate significantly different results based on a post-hoc Kruskal–Wallis test (P < 0.05). Data represent means ± SE. **(B)** IAA level (in ng per g fresh weight) in 3 week old WT (Torsdag), *rms1-2*, *rms2-1*, *rms3-2* and *rms4-1* plants treated hydroponically for 24 h with 0 or 3 μM (±)-3'-Me-GR24. Upper internodes below the apex (internode 8–9) and middle internodes (internode 4–5) were analysed (n = 4 pools of 8–10 plants each). Different letters indicate significantly different results between non-treated genotypes based on a Kruskal–Wallis test (P < 0.05). Asterisks denote significant differences between treated and control plants within a genotype based on a post-hoc Kruskal–Wallis test (P < 0.001). Data represent means ± SE.

If *RMS2* encodes an auxin receptor, the best candidate for the shoot-to-root RMS2-dependent feedback signal is auxin [54]. IAA is well known for its role in repressing CK biosynthesis [58,59,76] and stimulating SL biosynthetic gene expression [15,20,44]. Previous physiological characterization of the *rms* branching mutants showed that rather than being depleted in IAA levels, they often contained elevated IAA levels [48,66,77]. Therefore, a model can be proposed where the lack of SL response in the *rms* SL-biosynthesis and signalling mutants stimulates the synthesis of IAA, which controls CK levels in the xylem sap and SL biosynthesis gene expression via RMS2 (and possibly via other TIR1/AFB proteins). To test this model, we investigated whether SL treatment can repress IAA levels using the pea SL *rms* mutants. The *rms1-2* mutant (Torsdag background) was grown in a hydroponic system in the presence or absence of the synthetic SL analogue (±)-3'-Me-GR24. This analogue is more stable than the classical SL analog GR24 because of two methyl groups on the D-ring and strongly inhibits shoot branching in pea [78]. Analogues with this D-ring structure were shown to act via RMS3 with the same mechanism of perception as analogues with the canonical D-ring structure (present in natural SLs) with one methyl group at the 4′ position [26]. Levels of IAA were quantified in stem segments at upper, middle and basal nodes 6 h and 24 h after SL treatment. For all stem segments, *rms1-2* had higher IAA levels than WT (Fig 5A). IAA levels were reduced in the different stem segments within 6 h of (±)-3'-Me-GR24 application and were significantly decreased by 24 h (Fig 5A).

In a second experiment, IAA levels were quantified in stem segments at upper and middle nodes after 24 h of (±)-3'-Me-GR24 application in *rms1-2*, *rms2-1*, *rms3-2* and *rms4-1* in the Torsdag background. IAA levels were not reduced by (±)-3'-Me-GR24 application in both *rms3-2* and *rms4-1* SL response mutants but were decreased significantly in *rms1-2* and *rms2-1* (Fig 5B). A small IAA increase was observed in *rms4-1* upper stem segments after SL application, an opposite response to SLs regularly observed for *max2*/*rms4* that is not yet well understood [14,26]. These results indicate that SLs can repress IAA levels in the stem via *RMS3* and *RMS4*, and *RMS2* is not required for the regulation of IAA levels by SLs. Furthermore, *rms2* mutants can respond to SL (Fig 5B) to regulate both shoot branching and stem IAA content ([55]; S5 Fig). Therefore, the high IAA levels observed in the stems of SL biosynthesis (*rms1*, *rms5*) and response (*rms3*, *rms4*) mutants are at least in part due to impaired down-regulation of IAA biosynthesis in these mutants. The *rms2* mutant also contains very high levels of IAA in stems, particularly at upper nodes ([48,66]; Fig 5B and S6 Fig). To confirm that the high IAA stem levels of *rms2* is due to an impaired auxin response, rather than a lack of SL-mediated feedback suppression of IAA levels, IAA levels were quantified in the *rms1-1 rms2-2* double mutant (Parvus background) and compared to WT and single mutants. For both basal and upper internodes, IAA levels were higher in *rms1-1 rms2-2* than in WT and *rms1-1* and *rms2-2* single mutants (S6 Fig). This result indicates an additive effect of SL deficiency and a lack of auxin response on IAA content in the stem, similar to results reported with Arabidopsis *axr1* and *max1* mutants [79]. In Arabidopsis, a significant proportion of the auxin in stems is derived from active apices, including branches [79]. Accordingly, a strong gradient in IAA concentration along the Arabidopsis inflorescence stem is observed, with higher levels towards the stem base [32,80,81]. In contrast, in all our experiments on pea where different nodes along the stem were collected, the higher IAA levels were observed in the upper node below the apex indicating that the reduced IAA levels observed after SL application was not the result of reduced IAA export from active apices of axillary branches after SL application, at least in this upper node.

### Strigolactones rapidly down-regulate auxin biosynthesis gene transcript levels

To investigate the possible mechanism(s) of SL-mediated regulation of IAA levels, and the kinetics of the response, expression levels of key auxin biosynthetic genes were analyzed. In Arabidopsis, the TRYPTOPHAN AMINOTRANSFERASE OF ARABIDOPSIS1 (TAA1/TAR) enzymes convert tryptophan to indole-3-pyruvic acid (IPyA), which is then converted to IAA by the YUCCA (YUC) proteins [82,83]. The Pea RNA-Seq gene atlas (http://bios.dijon.inra.fr/FATAL/cgi/pscam.cgi; [68]) was used to select genes from these two families that were expressed in young shoots before flowering. Among the three *TAR* genes identified in pea, *TAR2* (JN990989 = PsCam045859) was selected for analysis as it is widely expressed whereas *TAR1* (JN990988 = PsCam038427) is specifically expressed in seeds and pods and *TAR3* (JN990990 = PsCam017219) is expressed in roots, nodules and seeds [84]. Several pea *YUC* genes are expressed in young shoots [85]. In a preliminary experiment, transcript levels of *TAR2*, *YUC1* and *YUC2* were analyzed in the upper internode (node 6-node 7) of WT^Torsdag^ and *rms1-2* (Torsdag background) mutant plants grown hydroponically with and without SLs ((±)-3'-Me-GR24., 3 μM) for 7 days. The SL biosynthesis gene *RMS5* (*PsCCD7*) was analyzed to confirm the effectiveness of the SL treatment. Transcript levels of the four genes followed the same pattern with high levels in *rms1* compared to WT and a significant decrease in *rms1* when grown with SLs (S7 Fig). *TAR2* and *YUC1* were chosen for further analysis together with *RMS5*.

**Fig 6:**
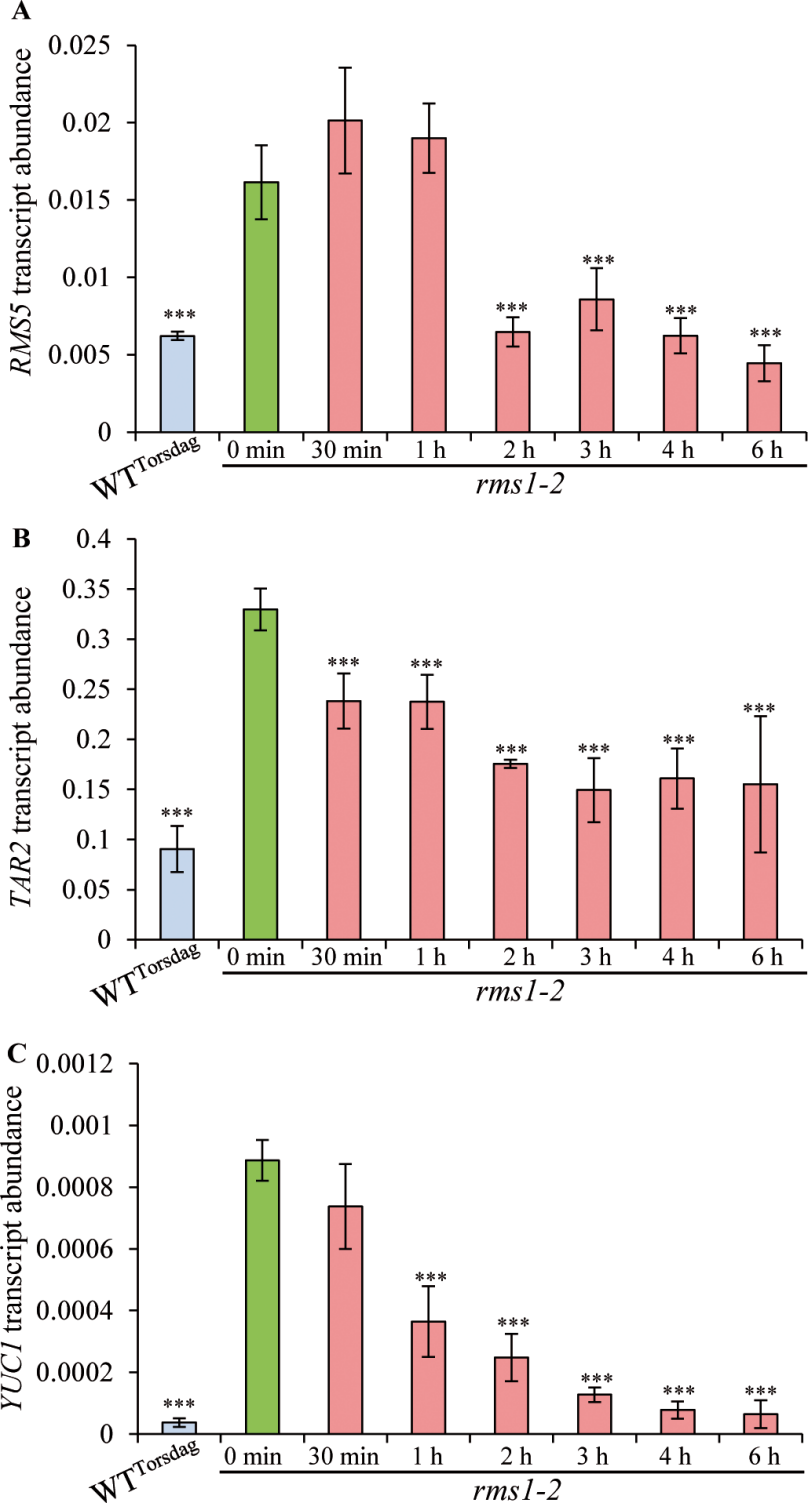
Strigolactone application down-regulates transcript abundance of auxin biosynthetic genes. *RMS5* **(A)**, *TAR2* **(B)** and *YUC1* **(C)** transcript abundance relative to *ACTIN* in internodes beneath the apex in 3 week old WT (Torsdag) and *rms1-2* plants treated hydroponically with (±)-3'-Me-GR24 (3 μM) for 0.5 h, 1 h, 2 h, 3 h, 4 h or 6 h; WT and *rms1* 0h samples were treated with control solution (n = 3 pools of 8–10 plants each). Asterisks denote significant differences between the treated and control *rms1* plants based on a post-hoc Kruskal–Wallis Dunnett test (P < 0.001). Data represent means ± SE.

Transcript levels of the selected genes were quantified after applying SLs for 0.5 h, 1 h, 2 h, 3 h, 4 h and 6 h to the SL-deficient mutant *rms1-2* (Torsdag background). As expected, transcript levels of *RMS5* were higher in *rms1* controls than in the WT and decreased significantly 2 h after SL treatment (Fig 6A). *TAR2* and *YUC1* followed the same pattern as *RMS5* with a significant decrease in *TAR2* and *YUC1* transcript levels after 30 min and 1 h, respectively (Fig 6B and 6C). Thus, SL treatment can significantly reduce *TAR2* transcript levels in upper internodes as soon as 30 min after treatment, before any significant decreases in *RMS5* transcript levels (after 2 h) and IAA levels (between 6 and 24 h) (Fig 5A). The reduction was not observed when using the *rms4* SL-response mutant (S8 Fig). Together these results suggest that SLs repress IAA levels in the stem at least in part by a rapid down-regulation of transcript levels of IAA biosynthesis genes. A more detailed tissue- and/or cell-specific IAA quantification could detect whether there is a significant decrease in IAA levels at earlier time points [86].

### Strigolactones down-regulate IAA levels in NPA treated plants

In Arabidopsis, *max* mutants display increased auxin transport in the primary inflorescence stem and increased PIN1 protein accumulation at the basal plasma membrane of xylem parenchyma cells, which can be rapidly reduced by GR24 treatment [40,41]. It is well known that IAA levels and polar auxin transport (PAT) are highly interconnected. To investigate whether the SL-mediated decrease in IAA levels could be mediated by the effect of SL on PAT, we tested if SL could still elicit a reduction in IAA levels in NPA-treated plants where PAT is severely compromised.

**Fig 7:**
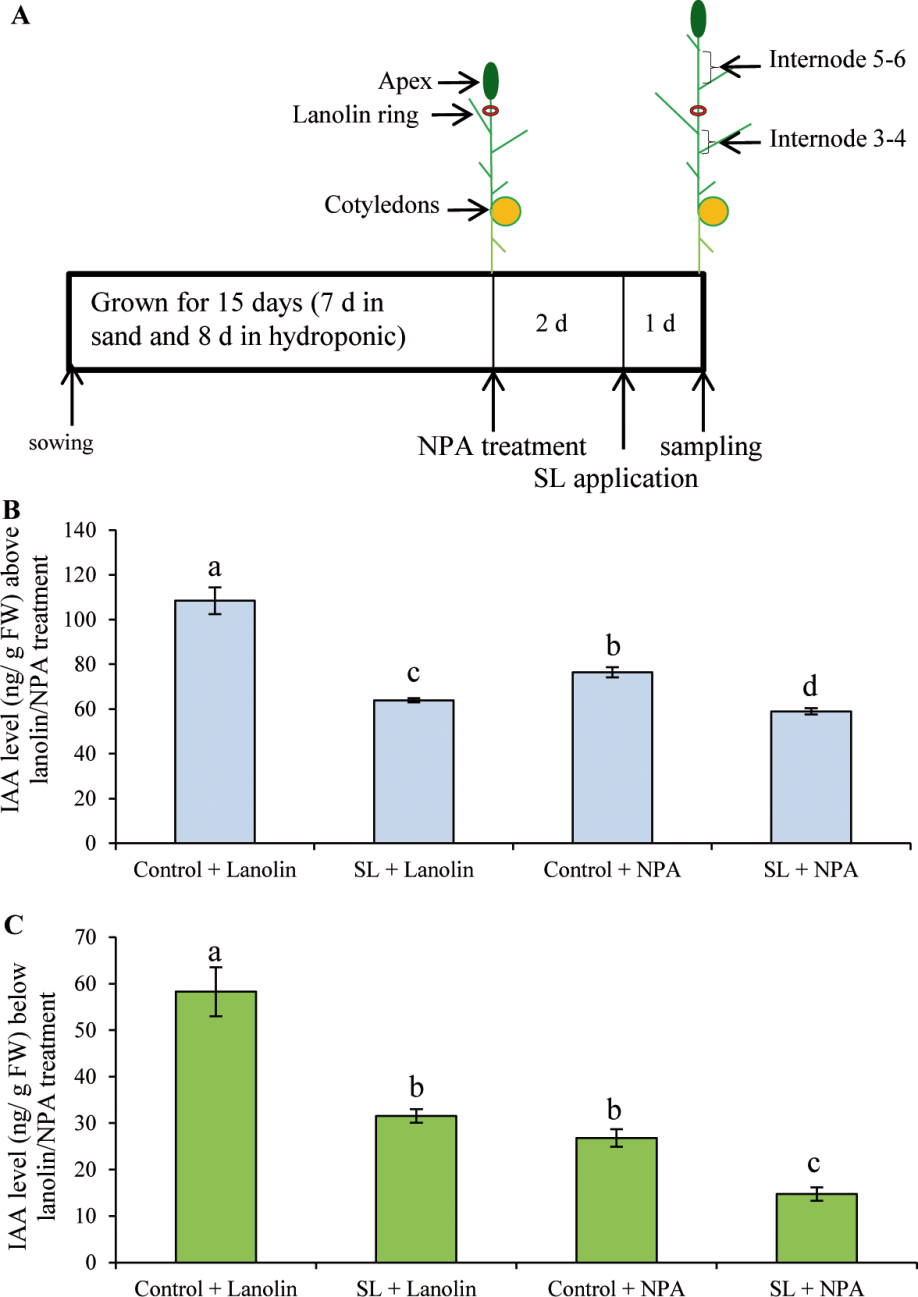
**Strigolactones down-regulate IAA levels in NPA treated plants (A)** Scheme of growth conditions and treatments. 15 day-old *rms1* plants were supplied with a lanolin ring, with 0% or 0.1% NPA, around the stem in the upper end of the oldest expanding internode (internode 4–5). Two days later, they were treated hydroponically with acetone or (±)-3'-Me-GR24 (3 μM) for 1 day. Internodes above (internode 5–6) **(B)** and below (internode 3–4) **(C)** the lanolin ring were harvested for IAA quantification (n = 4). IAA levels shown are in ng per g fresh weight. Different letters indicate significantly different results based on a post hoc Kruskal–Wallis test (P < 0.05). Data represent means ± SE.

15-d-old *rms1* plants were treated with a lanolin ring around the stem of the oldest expanding internode with or without 0.1% NPA. Two days later, (±)-3'-Me-GR24 was supplied hydroponically via the roots for 24 h, after which time the internodes above and below the NPA treatment site were harvested for IAA quantification (Fig 7A). The 3 day NPA treatment induced a strong decrease in IAA levels in internodes below (54% reduction) but also above the NPA lanolin ring (30% reduction) possibly due to reduced auxin export into the stem from leaves and to the systemic effect of NPA [87]. Below the site of lanolin/NPA treatment, the effect of SL on NPA treated plants was similar to that on lanolin treated control plants (45% and 46% reduction, respectively), although the absolute reduction was lower. Above the site of lanolin/NPA treatment, the SL effect on NPA treated plants was still significant but relatively smaller (23% reduction) compared to the effect of SL in lanolin treated control plants (41% reduction). These results suggested that SLs can decrease IAA levels in stems independently of their effects on polar auxin transport.

## Discussion

**Fig 8:**
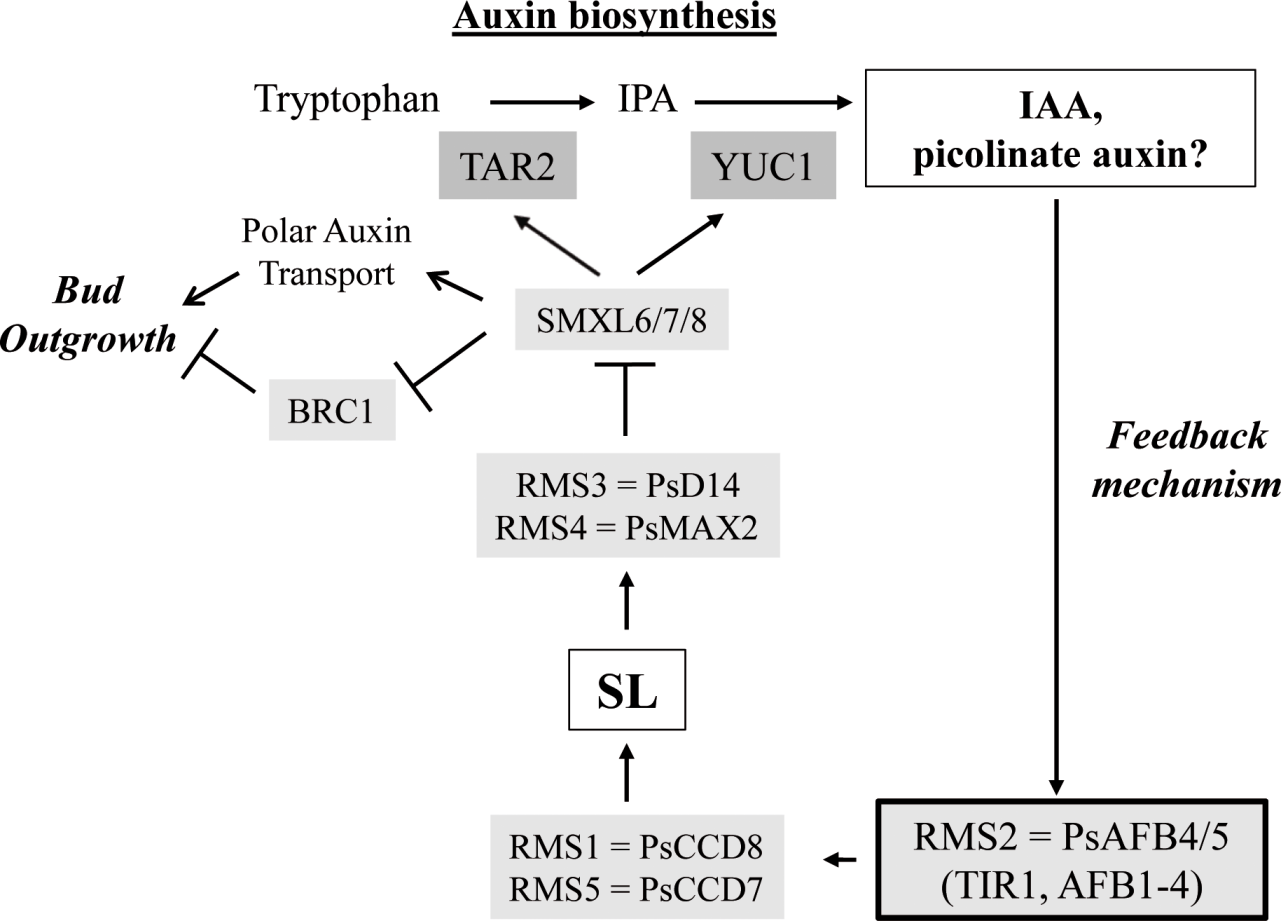
A proposed model for the SL biosynthesis negative feedback mediated by IAA and/or by an unknown endogenous “picolinate auxin”. SLs repress auxin biosynthesis by repressing the transcript levels of *TAR2* and *YUC1*; SLs act via RMS3, RMS4 and the SMXL6-8 proteins but not BRC1 for this regulation. The F-box proteins of the AFB4/5 clade of auxin receptors, together with other auxin receptors, mediate the auxin up-regulation of SL biosynthesis gene expression.

In several species, SL application down-regulates transcript levels of SL biosynthesis genes in the shoot, indicative of negative feedback control on SL biosynthesis [5,13,14]. In pea, a major contributor to this feedback is the *RMS2*-dependent shoot-to-root signal. Our findings suggest *RMS2* encodes the unique pea F-box protein of the AFB4/5 clade and yeast two- hybrid assays support the hypothesis that RMS2 functions as an auxin receptor (Figs 1 and 4). Together, this suggests the RMS2-dependent feedback signal is very likely auxin. Since all pea SL-defective mutants display high transcript levels of *RMS1* and low X-CK, it was proposed that a non-response to SL activated this shoot-to-root feedback signal [44,53,55]. Our demonstration that SL represses IAA levels in stems strengthens the hypothesis that auxin is an intermediate for the feedback control of SL biosynthesis, with SL repressing auxin biosynthesis and very likely auxin export from source tissues, and auxin(s) stimulating SL biosynthesis (Fig 8).

### RMS3/D14 and RMS4/MAX2, but not BRC1, are needed for SLs to repress IAA biosynthesis

Higher levels of stem IAA have been frequently observed in SL-defective mutants ([38,48,80,81,88], this work). Here we demonstrate that SLs decrease IAA levels in pea internodes with a significant reduction observed 24 h after SL feeding through the roots (Fig 5). When analyzing transcript levels of IAA biosynthesis genes in the internode below the apex, a significant reduction was observed within 30 min for *TAR2* and 1 hour for *YUC1* after SL application, whereas a significant decrease in the transcript levels of the SL biosynthesis gene *RMS5* was observed after 2 hours (Fig 6). These results indicate that the synthetic SL analog used in our experiments, or some derivative, was transported from root to shoot within 30 min (at a distance of at least 40 cm). The only SL transporter that has been identified to date is the PLEIOTROPIC DRUG RESISTANCE1 from *Petunia axillaris* (PDR1), a SL cellular exporter, expressed in root cortex and shoot axils [89]. To explain the rapid root-to-shoot long distance SL transport observed here, xylem transport seems more likely. Grafting experiments have clearly demonstrated that a wild-type rootstock can inhibit the shoot branching of an SL-deficient scion and that SL can only move in a root-to-shoot direction [46]. Recent work with SL root applications and SL analysis in shoot tissues has shown that root-to-shoot SL transport is a highly structure- and stereospecific process [90]. Currently it is unclear which SLs (non-canonical SL vs canonical SLs) or SL-derived metabolites are transported from the root to inhibit shoot branching. Nevertheless, some SL-related molecule can rapidly move to the shoot and decrease the expression of IAA biosynthesis genes in the stem. However, we cannot rule out additional effects of SL on other parts of the auxin synthesis and breakdown pathway to regulate the pool of IAA in the stem. SL-mediated repression of IAA biosynthesis has previously been proposed as a mechanism by which SL attenuates shoot gravitropism and tiller/branch angle in rice and in Arabidopsis [91].

In Arabidopsis, the synthetic SL analogue GR24 was shown to reduce PAT in the main stem by removing PIN1 from the plasma membrane by a rapid (<30 min), cycloheximide-independent, clathrin-dependent mechanism [40]. As IAA and PAT influence each other [92], we tested whether the decrease in IAA levels observed after SL application was due to the effect of SL on PAT. SL could still elicit reductions in IAA levels in NPA treated plants, suggesting that SLs also have an effect on auxin biosynthesis in stems that is mostly independent of PAT (Fig 7). The SL-triggered changes in stem IAA levels are therefore likely due to a combination of changes in auxin synthesis and changes in IAA export from young expanding leaves, which are a major source of stem auxin.

Using different mutants, we demonstrated that SLs reduce IAA levels via RMS3 and RMS4 but this action does not require RMS2 (Fig 5). The TCP transcription factor BRC1 is unlikely to be involved in this SL-mediated repression of auxin biosynthesis and *RMS1* expression [33]. Indeed, the high tillering rice *fc1* / *Osbrc1* mutant has normal *D10/OsCCD8* transcript levels [18] and normal IAA levels in shoot apices [17] in comparison to all SL-defective mutants. Similarly, the highly branched pea *Psbrc1* mutant has WT or lower *RMS1* transcript levels and in *Psbrc1* the profiles and absolute amounts of X-CKs are not significantly different from WT, whereas X-CK levels are very low in SL biosynthesis and response mutants [33,49,53]. In addition, Arabidopsis *brc1* mutants have normal stem auxin transport, further separating the activity of SL in modulating auxin homeostasis and *BRC1* expression [35,37].

In Arabidopsis, the SMXL6-8 proteins activate shoot branching, with SLs stimulating their ubiquitination and subsequent degradation via the proteasome [30,31]. In Arabidopsis seedlings, *MAX4* transcript abundance is higher than WT in *max3/Atccd7* and *max2* but lower than WT in *smxl6/7/8* triple mutants, *max3smxl6/7/8*, and *max2smxl6/7/8* quadruple mutant plants [30]. Therefore, when SMXL6-8 proteins are non-functional, the feedback signal is suppressed even in SL-defective mutant backgrounds where it is usually triggered. Furthermore, in *35S*:*SMXL6D-GFP* Arabidopsis plants bearing a dominant mutation conferring SL resistance equivalent to the rice d53 mutant, *MAX4* transcript levels are higher in comparison to WT and in *35S*:*SMXL6-GFP* transgenic plants [28,29]. Together, these results suggest the SMXL6-8 proteins are necessary for activating the feedback signal and the down-stream stimulation of SL-biosynthesis gene transcript levels. SMXL6-8 are also known to mediate the effects of SL on auxin transport [32]. Further studies with IAA quantifications in the recessive and dominant mutants for the SMXL6-8 proteins are necessary to confirm the involvement of these proteins in the regulation of IAA levels by SLs.

### Redundancy in the TIR1/AFB proteins in mediating the auxin-dependent feedback

As *RMS1* transcript levels are high in all SL-defective mutants and low in *rms2* epicotyls compared to WT, it was proposed that the *RMS2* gene is involved in the feedback control of SL biosynthesis [44,48,49,53]. However, double *rms* mutants with *rms2* have intermediate levels of *RMS1* transcripts and X-CK root export [20,43–45,49,53], suggesting *rms2* does not completely prevent feedback upregulation of *RMS1* expression and repression of X-CK [44,53]. A simple explanation for this is that other TIR1/AFB auxin receptors also play a role in mediating auxin-dependent feedback. In our experiments using Arabidopsis *afb* mutants, *afb4 afb5* double mutants have increased branching compared to WT. Yet, triple and quadruple mutants of the TIR1/AFB2 clade display a clear highly branched and dwarf phenotype in Arabidopsis [93] and downregulation of *OsTIR1* and *OsAFB2* results in higher tiller numbers in rice [94]. Higher shoot branching and low *MAX3/MAX4* expression is also observed in auxin-related mutants such as *axr1* or the semi-dominant *bodenlos* (*bdl*) mutant containing a mutation that stabilizes the IAA12 protein [15]. Together these results suggest that members of the AFB4/AFB5 clade are unlikely to be the only auxin receptors specifically involved in mediating the auxin-dependent negative feedback in shoot branching regulation. It may also explain why the *rms2* mutant responds to IAA in some assays, e.g. *RMS1* transcript levels are increased after IAA application but do not attain the high levels observed in the SL-response *rms3* and *rms4* mutants [44].

### Activation of the auxin-dependent feedback signal by shoot branching

The elevated IAA levels observed in SL defective mutants may also result from auxin exported from the branches and entering the main stem and this auxin likely also participates in feedback up-regulation of SL biosynthesis genes [32,55,80]. Indeed, a strong IAA concentration gradient which increases towards the stem base is observed in Arabidopsis, particularly in highly branched mutants [32,80,81]. This gradient is thought to be due to the increased number of active apices exporting IAA into the basal stem combined with their increased auxin transport activity [32,80]. Interestingly, an opposite IAA gradient was found in pea, with higher IAA concentrations in the upper internode; IAA levels in this internode are even higher than those in the apical part of the shoot [77]. This pattern is also observed in highly branched mutants ([48,77]; this work). The origin of these opposing IAA gradients is not clear and requires further investigation. They may be due in part to different experimental systems and/or developmental stages, as shoot branching is generally analyzed before floral transition in pea, versus after floral transition in Arabidopsis [95].

The higher IAA level in the upper internode of *rms* mutants suggests that, at least in pea, it is the non-response to SL (possibly mediated by high levels of SMXL proteins), rather than the high shoot branching *per se*, which activates the biosynthesis of auxin and feedback up-regulation of SL biosynthesis gene transcript levels. This hypothesis was already tested using the *s**uppressed* *ax**illary meristem1* (*sax1*) mutation which inhibits the formation of axillary meristem at most nodes [53,96]. Grafting experiments showed that X-CK was similarly reduced when WT rootstocks were grafted to either *rms4* single mutant or *rms4 sax1* double mutant scions, despite having strongly reduced branching in *rms4 sax1* shoots due to the absence of most axillary meristems [53]. Another example where high IAA levels are not related to high shoot branching is the maintenance of high IAA levels in *rms2* shoots even when branching is suppressed by grafting to WT rootstocks [66]. This observation supports the hypothesis that the high IAA content of *rms2* is due to its non-response to auxin. A shoot-derived signal, very likely IAA, has also been proposed in pea, as a transient increase in *RMS1* transcript levels occurs in *rms2* epicotyls only when a strong basal branch is growing above it [55]. These data suggest that *rms2* can respond to this lateral branch-derived signal. Interestingly, the *rms4* mutant showed *RMS1* transcript levels similar to WT in the internode below the apex, where IAA levels are also comparable to WT (Fig 5B), whereas constitutive elevated *RMS1* transcript levels in *rms4* epicotyls are observed [55]. These data demonstrate the dynamic spatio-temporal control of auxin-SL feedback which necessitates careful analysis of phytohormone distribution [86].

Low SL levels in shoots have hampered efforts to perform direct quantifications in aerial tissues. The low *RMS1* expression in *rms2* epicotyls suggests that the mutant is highly branched in part because of reduced SL production [55]. However, quantifications of the three main SLs found in pea (orobanchol, fabacyl acetate, orobanchyl acetate) in root exudates and root tissues of *rms4* and *rms2* under low phosphate conditions are similar to those in WT [97]. Nevertheless, branching in *rms1* shoots can be completely rescued by WT rootstocks, but only partially by *rms2* rootstocks [48]. This suggests that *rms2* mutants are highly branched at least in part due to reduced SL production. The strong additive branching phenotype of the *rms1 rms2* double mutant compared to the single mutants [48] indicates that another component, possibly the high CK levels measured in both *rms2* shoot tissues and X-CK, also contributes to the increased shoot branching of *rms2*. In Petunia, comparison of *DAD1/PhCCD8* and *DAD3/PhCCD7* transcript levels in *dad* roots and stems shows elevated *PhCCD7*/*8* gene expression in stems relative to WT, but not in roots [16,52]. Thus, there is likely to be complex and precise regulation of SL biosynthetic gene expression and SL levels in different tissues. Another layer of regulatory complexity is added by the homeostastic regulatory mechanisms controlling auxin levels, as demonstrated by the recent discovery of the DIOXYGENASE FOR AUXIN OXIDATION1 (DAO1) which catalyzes the oxidation of IAA into 2-oxindole-3-acetic acid (oxIAA) [98–100].

### Conclusion

Here we demonstrated that *RMS2* encodes a member of the TIR1/AFB family of auxin receptors of the AFB4/5 subclade. We confirmed the role of auxin in mediating the negative feedback of SL and propose a model where auxin and SLs regulate each other’s metabolism, and the distribution of auxin is dynamically controlled by growing branches and polar transport. We cannot rule out that other mechanisms are involved in the negative feedback of SL. A direct SL-feedback mechanism may be identified when the function and targets of the SMXL proteins are better understood. Moreover, the selectivity of AFB4/AFB5 in picolinate auxin perception, conserved between Arabidopsis and pea, and the maintenance during evolution of the AFB4/5 sub-clade, are quite intriguing; a specific endogenous ligand for AFB4/5 may yet be discovered [101]. Better clarity of the SL feedback mechanism(s) at play may be gained through further analysis of such a ligand and the affinities of particular IAAs to AFB4/5.

## Materials and methods

### Plant material and growth conditions

The pea (*Pisum sativum*) branching *rms1-1* (WL5237), and *rms2-2* (WL5951) mutants were derived from the tall line Parvus. The *rms1-1 rms2-2* double mutant was kindly given by Christine Beveridge (University of Queensland, AU). The *rms2-1* mutant (K524), the *rms3-2* mutant (K564), and the *rms4-1* mutant (K164) were obtained in the tall line Torsdag. The *rms1-10* (M3T-884) and *rms4-3* (M3T-946) were obtained from the dwarf cv Térèse. All these mutants were described in [66,71,102]. The *rms1-2* (Torsdag) mutant line was obtained by backcrossing the *rms1-2* allele from the line WL5147 derived from Weitor; [48] into the WT line Torsdag three times. The *rms2-1* mutant in Térèse background was obtained by backcrossing the *rms2-1* allele (from the K524 line in Torsdag background) into the WT line Térèse seven times. The mapping population was an F2 of 528 individuals derived from the cross (K524 x JI281). The parental line JI281 has been used previously to generate a molecular map of pea [67] and was obtained from JIC Norwich-UK (http://www.jic.bbsrc.ac.uk/germplas/pisum/index.htm).

All Arabidopsis (*Arabidopsis thaliana*) lines used were in the Col-0 background and mutant lines for *max2-1* [103], *max4-1* [19], *tir1-1* [104], *afb2-3* [105], *afb4-8*, and *afb5-5* [73] were previously published.

Pea plants were grown in glasshouse (23°C day/ 15°C night) under a 16-h photoperiod (the natural daylength was extended or supplemented during the day when necessary using sodium lamps) in pots filled with clay pellets, peat, and soil (1:1:1) supplied regularly with nutrient solution (140 mg/l N, 76 mg/l P_2_O_5_, 231 mg/l K_2_O, 146 mg/l CuO, 15 mg/l MgO; 0.82 mg/l K/(Ca + Mg); 3.64 mg/l NO_3_^-^/NH_4_^+^, diluted 200 times in water). Nodes were numbered acropetally from the first scale leaf as node 1. Arabidopsis plants were cultivated in a glasshouse or growth chamber (20°C day and night) under a 16-h photoperiod and humidity 70%.

### RMS2 locus positional cloning

The F2 population of 528 individuals were phenotyped for *RMS2* and genotyped for molecular markers designed on the basis of the conservation of synteny between the pea and *Medicago truncatula* genomes (http://jcvi.org/medicago/). Putative pea orthologues of *M*. *truncatula* genes located on chromosome 5 in the vicinity of genes involved in hormone signaling were identified *in silico* in the transcriptome databases of NCBI (expressed sequence tags, transcriptome shotgun assembly). In order to identify polymorphisms, the corresponding genomic sequences were PCR amplified and sequenced in both parents of the mapping population. The mapping population was then genotyped using CAPS (cleaved amplified polymorphic sequences) assays or sequencing. Primers and polymorphisms used for these markers are given in Table 1. For sequencing *PsAFB4/5* in WT and mutant lines, the primers used are given in Table 2.

**Table 1:** Primers and polymorphism used for the pea markers.

Marker	Primers	Sequence	Markers
PsAFB4/5	For- PsAFB4/5	GGATGAGGGTTTTGGTGCTA	*Mse*1 digestion site in K524 (198 + 91 bp)
	Rev-PsAFB4/5	ACAGGACGACATCCAAAGGA	
PsU81288	For-U81288	GCTTCTCTATTAGCCGCACTTG	Length polymorphism : 630 bp in K524, 545 bp in JI281
	Rev-U81288	CTGACCACTCCCATCGTTTT	
PsFG536326	For- FG536326	AGTTGAAGTAGAAGTGGT	Length polymorphism : 178 bp in K524, 164 bp in JI281
	Rev- FG536326	AAATCTCGGAATGATGCAATC	
PsAM161737	For- AM161737	AAGACTTGGCAATTTCAAA	*Mse*1 digestion site in JI281 (57+139 bp)
	Rev- AM161737	CTATGACATAAACGGGATGC	
PsFG535768	For- FG535768	CGCATGAACAGAAAGAGAAGC	Sequencing
	Rev- FG535768	CAACCACATGGAAGAAATTAATCA	
49547	For- 49547	GTGGTTCCAGTTCAACAAGG	Sequencing
	Rev- 49547	TGCTTTCTCTGCCACTGAAG	
18665	For- 18665	GAGGAACATGAAGAGGAGATGG	*Taqα*1 digestion site in JI281 (73 + 241 + 448 bp)
	Rev- 18665	GGGTTTCTTACCAAGAGTGTTC	
9581	For-9581	ATTCAGATCTCAACACAGGTA	Length polymorphism : 190 bp in Tor, 201 bp in JI281
	Rev-9581	AAATCTGTTCTTGGATATAGA	
20620	For-20620	TGAAAGCGAGATGCATGAAG	*Hae*III digestion site in JI281 (218 + 651 bp)
	Rev-20620	AGAGGCGTGCACTCTTGTTT	
20123	For-20123	CGGTGGTGGTTCAGTCTTTC	*Tsp45R*I digestion site in JI281 (428 + 393 bp)
	Rev-20123	TTGCTTCCCCATATCAAAGG	

**Table 2:** List of primers and their sequences used to sequence the pea RMS2 gene.

Primers	Sequence 5′ 3′
*PsRMS2*_122_F	CCATCTCTCTCTCACTCCCATTT
*PsRMS2*_493_R	GGCGTTGCGGTCTTTGCG
*PsRMS2*_337_F	CGCCGAATCATCCACCTC
*PsRMS2*_928_R	CATCCTCAACCTCAATCACAG
*PsRMS2*_788_F	TCGTTCGTCGGTTTTCGTGA
*PsRMS2*_1704_R	GCACCAAAACCCTCATCC
*PsRMS2*_1621_F	TCTTGTGGTGTTTCGTCTCT
*PsRMS2*_2350_R	TTCTCTCCCCTCCTCTCC
*PsRMS2*_1479_F	CGAGGGAGGAAACTGAAG
*PsRMS2*_2146_R	CTACTGCAGAATGGTAAC

### Strigolactone application

Pea hydroponic culture was done as described in [78] using 33L-polyvinyl chloride opaque pots. The hydroponic culture solution was continuously aerated by an aquarium pump and was replaced weekly. Acetone or (±)-3′-Me-GR24 (dissolved in acetone) (kindly provided by F.D. Boyer, Institut de Chimie des Substances Naturelles) were added to the hydroponic culture solution to give a final concentration of 0 or 3 μM of (±)-3′-Me-GR24 and 0.01% acetone. (±)-3′-Me-GR24 corresponds to compound 23 in [78].

### Picloram treatment

After 2 weeks, plants were sprayed with control solution (water, 4% ethanol) or with a solution of picloram (4-amino-3,5,6-trichloro-2-pyridinecarboxylic acid) at 0.83 mM (200 g/ha) or 2.07 mM (500 g/ha). Chlorophyll content was estimated with a SPAD (Soil Plant Analysis Development) chlorophyll meter (Minolta, SPAD-502 model, Tokyo, Japan) after 8 days on the stipule at node 6 (2 repetitions per stipule on 8–12 plants).

### *In vitro* IAA treatment

The *in vitro* IAA treatment of isolated internodes was adapted from [106]. Internodes 4–5 was harvested from 16-d-old plants and incubated in buffer or buffer supplemented with IAA (10 μM). After 3 h, the internodes were collected for RNA extraction (3 biological repeats of 6 internodes).

### RNA extraction and cDNA synthesis

RNA extraction and cDNA synthesis were adapted from [33]. Total RNA was isolated from 8 to 10 pea internodes or 10 to 15 Arabidopsis basal stem using TRIZOL reagent (Invitrogen) following the manufacturer’s protocol. DNase treatment was performed to remove DNA using the Qiagen RNase-Free DNase Set (79254) and the RNeasy Mini Kit (74904) and eluted in 50 mL of RNase-free water. RNA was quantified using NanoDrop 1000 and migrated on gels to check RNA non-degradation. The absence of contamination with genomic DNA was checked using 35 cycles of PCR with *RMS1* primers (5′-GGA ATG GTC CGG GCATGT G-3′ and 5′-TGA GAC TCG ATC TGC CGG TGA-3′). Total cDNA was synthesized from 2 μg of total RNA using 50 units of RevertAid H Moloney murine leukemia virus reverse transcriptase in 30 μL following the manufacturer’s instructions with poly(T)18 primer. cDNA was diluted 10 times before subsequent analysis.

### qPCR and oligonucleotides

Quantitative reverse transcription-PCR analyses were adapted from [33]. They were performed using SsoAdvanced Universal SYBR Green SuperMix (Biorad). Cycling conditions for amplification were 95°C for 10 min, 50 cycles of 95°C for 5 s, 62°C for 5 s, and 72°C for 15 s, followed by 0.1°C s–1 ramping up to 95°C for fusion curve characterization. Three biological repeats were analyzed in duplicate. To calculate relative transcript levels, the comparative cycle method based on non-equal efficiencies was used [107]. Transcript levels for the different genes were expressed relative to the expression of the *PsACTIN* gene for pea and of the *AtAPT* gene for Arabidopsis.

For qPCR in Arabidopsis, the following oligonucleotides were used: *AtAPT*, 5′-CGG GGA TTT TAA GTG GAA CA-3′ and 5′-GAG ACA TTT TGC GTG GGA TT-3′; *AtMAX3*, 5′-TCG TTG GTG AGC CCA TGT TTG TC-3′ and 5′-TCT CCA CCG AAA CCG CAT ACT C-3′ [108]. For qPCR in pea, the following oligonucleotides were used: *PsACTIN*, 5′-GTG TCT GGA TTG GAG GAT-3′ and 5′-GGC CAC GCT CAT CAT ATT-3′; *PsRMS5*, 5′-TGA CCG ACG GTT GTG ATT TGG-3′ and 5′-GCG GCA TCT TAA AGA CTC CGT AC-3′; *PsYUC1*, 5′-TTG CTA CCG GTG AAA ATG CTG A-3′ and 5′-CAT GAA AAT GTT CCA TAC CAT GAA TC-3′; *PsYUC2*, 5′- AGA GAA TGC CGA GGC TGT TGT G-3′ and 5′-AAG TTC CAT TCC AGA ATT TCC ACA TCC AA-3′ [85]; *PsTAR2*, 5′- TGG TGA ACC GTG GTG CAT TG-3′ and 5′- GCT GGT TGA GGT TCC AAC ACC TG-3′ [84].

### IAA quantification

IAA was extracted from 100 mg of fresh powder per sample with 0.8 mL of acetone/water/acetic acid (80/19/1 v:v:v). Indole-3-acetic acid stable labelled isotopes were prepared and used as internal standards (2 ng/sample) as described in [109]. The extract was vigorously shaken for 1 min, sonicated for 1 min at 25 Hz, shaken for 10 minutes at 4°C in a Thermomixer (Eppendorf®, and then centrifuged (8,000g, 4°C, 10 min.). The supernatants were collected, and the pellets were re-extracted twice with 0.4 mL of the same extraction solution, then vigorously shaken (1 min) and sonicated (1 min; 25 Hz). After the centrifugations, the three supernatants were pooled and dried (Final Volume 1.6 mL).

Each dry extract was dissolved in 100 μL of acetonitrile/water (50/50 v/v), filtered, and analyzed using a Waters Acquity ultra performance liquid chromatograph coupled to a Waters Xevo Triple quadrupole mass spectrometer TQS (UPLC-ESI-MS/MS). The compounds were separated on a reverse-phase column (Uptisphere C18 UP3HDO, 100*2.1 mm*3μm particle size; Interchim, France) using a flow rate of 0.4 mL min-1 and a binary gradient: (A) acetic acid 0.1% in water (v/v) and (B) acetonitrile with 0.1% acetic acid. The column temperature was 40°C. We used the following binary gradient (time, % A): (0 min., 98%), (3 min., 70%), (7.5 min., 50%), (8.5 min., 5%), (9.6 min., 0%), (13.2 min., 98%), (15.7 min., 98%). Mass spectrometry was conducted in electrospray and Multiple Reaction Monitoring scanning mode (MRM mode), in positive ion mode. Relevant instrumental parameters were set as follows: capillary 1.5 kV (negative mode), source block and desolvation gas temperatures 130°C and 500°C, respectively. Nitrogen was used to assist the cone and desolvation (150 L h^-1^ and 800 L h^-1^, respectively), argon was used as the collision gas at a flow of 0.18 mL min^-1^.

### Yeast two hybrid (Y2H) assays

Y2H assays were carried out as in [75,110]. The plasmids pGILDA-TIR1, pB42AD-IAA7, pB42AD-IAA3, and pB42AD-ASK1 were described previously [75,110]. The mutant iaa7 construct with three substituted residues in the degron was produced by site directed mutagenesis of the pB42AD-IAA7 plasmid with the primers 5′-GCT AAA GCA CAA GTG GTG AGA TGG TCA TCT GTG AGG AAC TAC AGG A-3′ and 5′-TCC TGT AGT TCC TCA CAG ATG ACC ATC TCA CCA CTT GTG CTT TAG C-3′. WT and mutant RMS2 cDNA sequences were amplified using primers 5′-GGG GAC AAG TTT GTA CAA AAA AGC AGG CTT C**AT G**AG AGA AAA CCA TCC TCC-3′ (start codon in bold and attB1 site underlined) and 5′-GGG GAC CAC TTT GTA CAA GAA AGC TGG GTC TCA **CTA** CTG CAG AAT GGT AAC AT-3′ (STOP codon in bold and attB2 site underlined) and recombined into pDONR207 using BP Clonase (Invitrogen) then recombined into a Gateway-compatible version of pGILDA using LR Clonase II (Invitrogen). Site-directed mutagenesis of pDONR207 containing RMS2 coding sequence was performed using the QuickChange II XL Site Directed Mutagenesis kit (Stratagene) and primers 5′-CCT AAT TTG CAG AAA CTT AAA ATC AGG GAC AGT CCC TTC GGG G-3′ and 5′-ACA TCA AGT CGG TTA CCG TCA AGA GAA AAC CTA GGT TTG CGG ATT-3′ to obtain the *rms2-1* and *rms2-2* mutant sequences, respectively. AtAFB5 was amplified using the primers 5′-CAC CAT GAC ACA AGA TCG CTC AGA AAT-3′ and 5′-TAA AAT CGT GAC GAA CTT TGG TG-5′ and cloned into pENTR D/TOPO (Invitrogen), and a Myc tag added by ligating a double-stranded oligo (5′-CGC GAA CAG AAA CTG ATC TCT GAA GAA GAT CTG TAG-3′ plus 5′-CGC GCT ACA GAT CTT CTT CAG AGA TCA GTT TCT GTT-3′) into the *Asc*I restriction site. The resulting entry clone and a Myc tag only control were recombined into the same pGILDA-derived destination vector to produce the pGILDA-AtAFB5-Myc and pGILDA-Myc (control) plasmids, respectively. Yeast strain EGY48 [p8Op:lacZ] was co-transformed with one pGILDA plasmid and one pB42AD plasmid and transformants selected on a medium lacking uracil, histidine, and tryptophan. Independent transformants were cultured and dilutions spotted on SD/Gal/Raf/X-Gal plates with or without IAA. The lexA-RMS2, lexA-rms2-1 and lexA-rms2-2 proteins were expressed to similar levels based on detection on a Western blot using an anti-lexA antibody (Millipore, 06–719) (S4 Fig).

### Software for statistical analysis and for phylogenetic trees

Statistics were performed with the software R and MEGA7 was used for phylogenetic trees [111].

## Supporting information

S1 Fig Protein sequence alignement of pea and Arabidopsis TIR1/AFB auxin receptor family. Protein sequences were obtained from TAIR ® for Arabidopsis and from the Pea RNA-seq Gene Atlas (http://bios.dijon.inra.fr/FATAL/cgi/pscam.cgi) for pea. Position of leucine rich repeat domains (LLR) (in blue) and F-box domain (in green) were obtained by NCBI ® web-interface. Position of 5 essential amino acids which form the auxin binding pocket of TIR1 are indicated in pink according to [70]. For AtAFB4, AtAFB5 and PsAFB4/5 (RMS2), His78 and Ser438 were not conserved. Specific N-terminal domain (in grey) from AtAFB4 and AtAFB5 [91] is also present in pea PsAFB4/5 (RMS2) protein. Position of *rms2-1* (E532K) and *rms2-2* (G117R) mutations are indicated in purple.
(TIF)

S2 Fig The transcriptional response of SL biosynthesis genes is strongly reduced in *rms2* mutants. Transcript levels of *RMS1* **(A)** and *RMS5* **(B)** and a pea homologue of the rice *D27* gene, PsCam038200 (*PsD27*) **(C)** relative to *ACTIN* in isolated pea internodes of 16 d old plants treated *in vitro* with 0 or 10 μM IAA for 3 h (n = 3 pools of 6 internodes 4–5 from 6 plants. Asterisks denote significant differences between IAA-treated and control-treated stems based on a post-hoc Kruskal–Wallis test (P < 0.001); (x 2) above IAA-treated stems refers to 2-fold change in comparison to control-treated stems. Different letters indicate significantly different results between control-treated genotypes based on a post-hoc Kruskal–Wallis test (P < 0.05). Data represent means ± SE. **(D)** Phylogenic tree of D27 proteins from rice (Os), Arabidopsis (At), pea (Ps), *Vitis vinifera* (Vv) and *Physcomitrella patens*. Protein sequences were aligned and used to generate the Maximum Likelihood phylogenetic tree with 1,000 bootstrap replicates. The percentage of replicate trees in which the associated taxa clustered together in the bootstrap test are shown next to the branches. Analyses were conducted in MEGA7.
(TIF)

S3 Fig *rms2* pea mutants are picloram resistant. SPAD values (determined with a Soil Plant Analysis Development chlorophyll meter), 8 days after spraying 2 week old plants with ~3 ml of 0.83 mM picloram solution (n = 8). Asterisks denote significant differences between the mutant and the corresponding WT plants, or between the treated and the non-treated plants in WT Cameor, based on a post-hoc Kruskal–Wallis test (P < 0.001). Data represent means ± SE.
(TIF)

S4 Fig Western blot analysis for RMS2 protein expression in the Y2H assay. **(A)** Representative western blot to detect lexa-RMS2 level analyzed by immunoblot using α-LEXA. Protein extracts from yeast were separated by 10% SDS-PAGE and identified as approximately 90 KDa bands were detected as expected for LexA–RMS2 fusions. **(B)** Ponceau staining is included for loading reference.
(TIF)

S5 Fig *rms2* is not affected in SL response. Total branch length was measured in 2 week-old WT (Torsdag background), *rms1-2* and *rms2-1* plants after 1 week of hydroponic treatment with (±)-3'-Me-GR24 (3 μM) or control solution (n = 12). Asterisks denote significant differences between treated and corresponding control plants based on a post-hoc Kruskal–Wallis test (P < 0.001). Data represent means ± SE.
(TIF)

S6 Fig Effects of *rms1* and *rms2* on IAA levels in shoots are additive. IAA levels (ng per g fresh weight) in upper internodes below the apex (internode 6–7) and basal internodes (internode 3–4) of 20 day old WT (Parvus), *rms1-1*, *rms2-2* and *rms1-1 rms2-2* double mutant plants (n = 4 pools of 10–12 plants). Different letters indicate significantly different results between genotypes based on a post hoc Kruskal–Wallis test (P < 0.05) in basal internode (lowercase), or in upper internode (capital letters). Data represent means ± SE.
(TIF)

S7 Fig Strigolactone application down-regulates transcript abundance of auxin biosynthetic genes. *RMS5* **(A)**, *TAR2* **(B),** *YUC1* **(C)** and *YUC2* **(D)** transcript abundance relative to *ACTIN* in internodes below the apex of 3 week-old WT (Torsdag) and *rms1-2* plants treated hydroponically with (±)-3'-Me-GR24 (3 μM) or control solution for 7 days (n = 3 pools of 8–10 plants). Asterisks denote significant differences between treated and corresponding control plants based on a post-hoc Kruskal–Wallis test (P < 0.001). Data represent means ± SE.
(TIF)

S8 Fig Strigolactone application down-regulates transcript abundance of auxin biosynthetic genes via RMS4. *RMS5* **(A)**, *TAR2* **(B)** and *YUC1* **(C)** transcript abundance relative to *ACTIN* in internodes 5–6 of 14 d old plants treated hydroponically with (±)-GR24 (3 μM) or control solution for 24 h (n = 3 pools of 8 plants). Asterisks denote significant differences between treated and corresponding control plants based on a post-hoc Kruskal–Wallis test (P < 0.001). Different letters indicate significantly different results between non-treated genotypes based on a Kruskal–Wallis test (P < 0.05). Data represent means ± SE.
(TIF)
