# Supplementary figures and images for: The pea branching RMS2 gene encodes the PsAFB4/5 auxin receptor and is involved in an auxin-strigolactone regulation loop
Source: PLoS Genet. 2017 Dec 8;13(12):e1007089. doi: 10.1371/journal.pgen.1007089 (PMC5738142; doi:10.1371/journal.pgen.1007089)

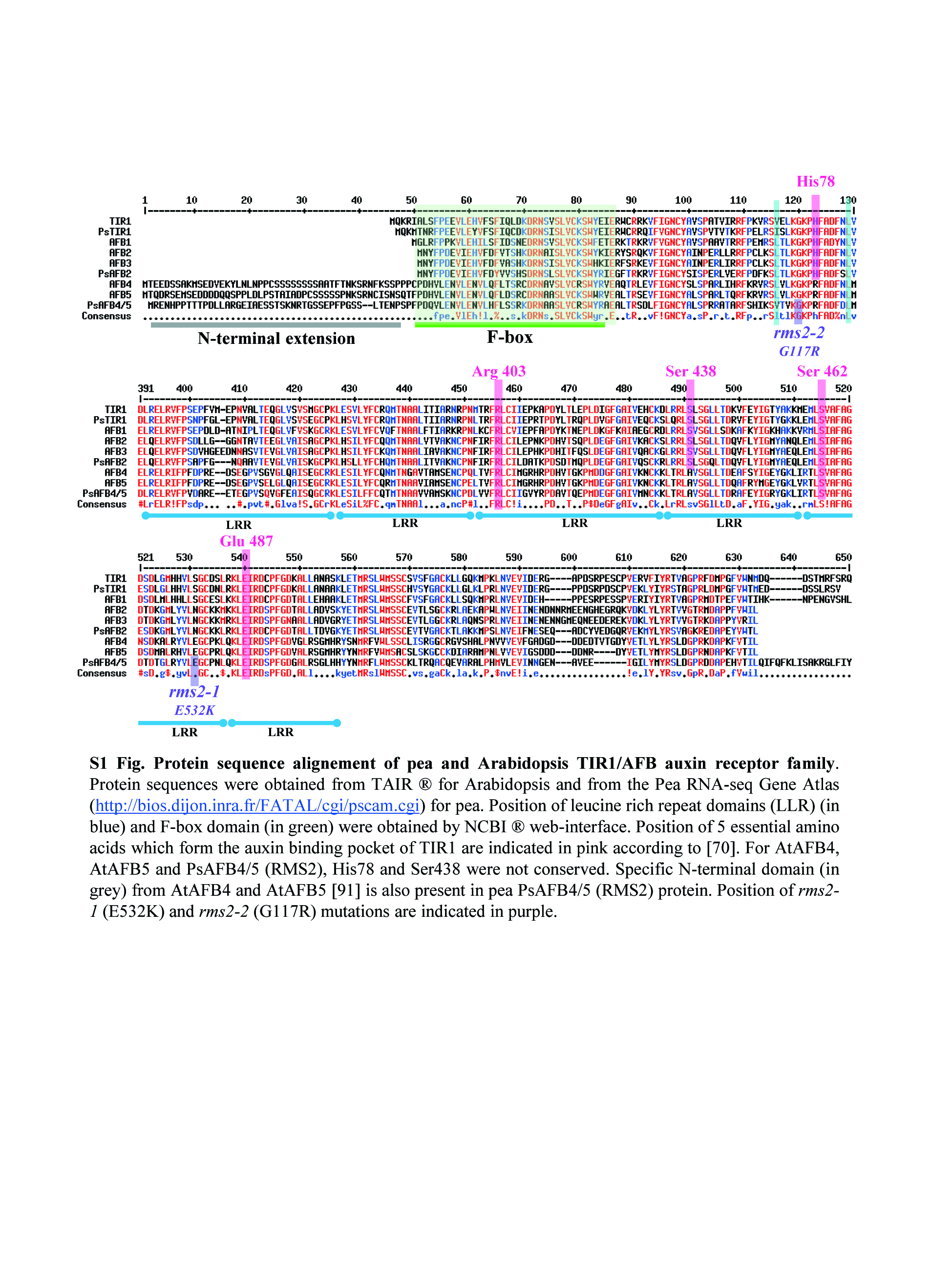

Supplement: S1 Fig — Protein sequences were obtained from TAIR ® for Arabidopsis and from the Pea RNA-seq Gene Atlas (http://bios.dijon.inra.fr/FATAL/cgi/pscam.cgi) for pea. Position of leucine rich repeat domains (LLR) (in blue) and F-box domain (in green) were obtained by NCBI ® web-interface. Position of 5 essential amino acids which form the auxin binding pocket of TIR1 are indicated in pink according to [70]. For AtAFB4, AtAFB5 and PsAFB4/5 (RMS2), His78 and Ser438 were not conserved. Specific N-terminal domain (in grey) from AtAFB4 and AtAFB5 [91] is also present in pea PsAFB4/5 (RMS2) protein. Position of rms2-1 (E532K) and rms2-2 (G117R) mutations are indicated in purple. (TIF) [file pgen.1007089.s001.tif]

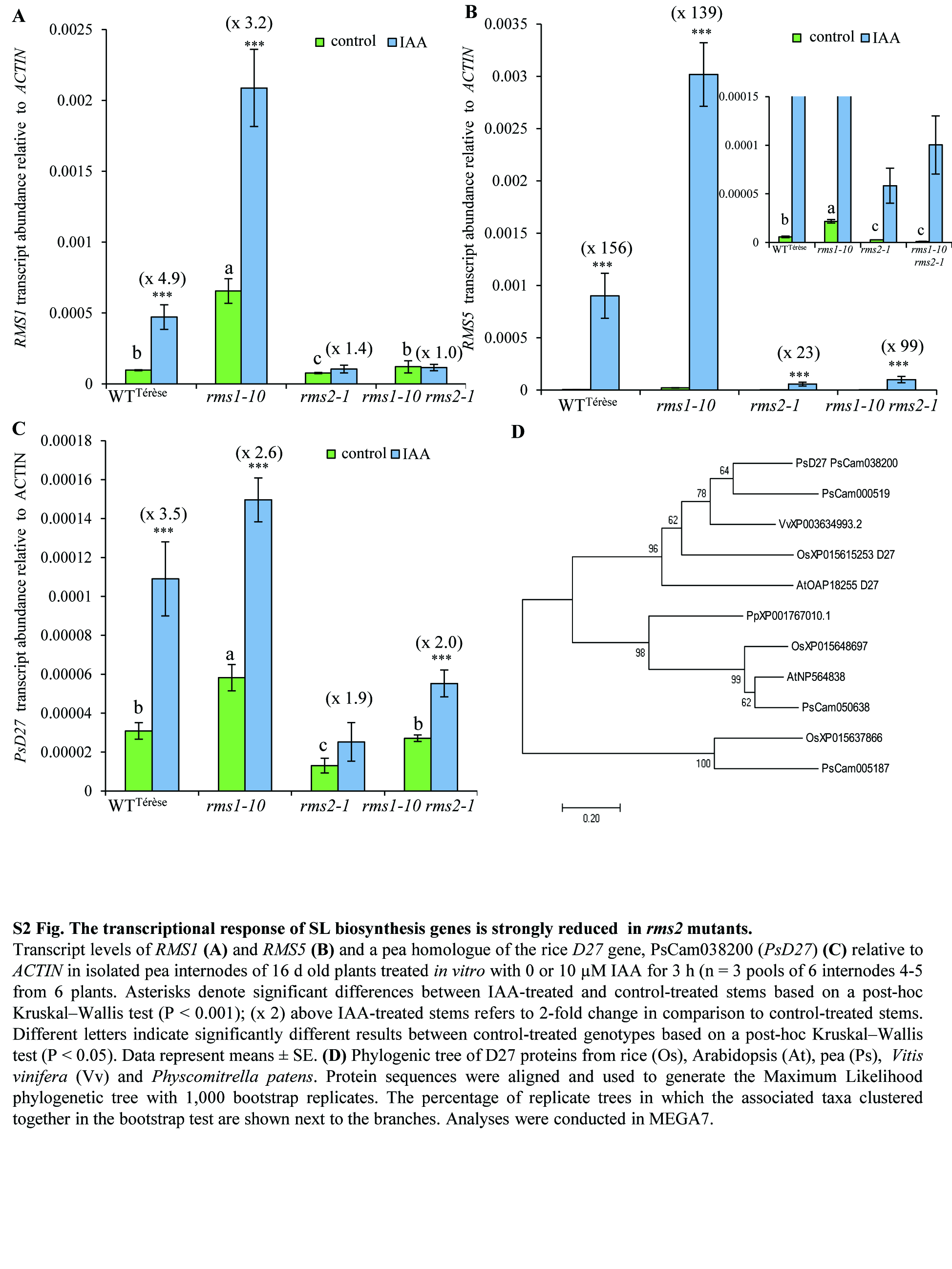

Supplement: S2 Fig — Transcript levels of RMS1 (A) and RMS5 (B) and a pea homologue of the rice D27 gene, PsCam038200 (PsD27) (C) relative to ACTIN in isolated pea internodes of 16 d old plants treated in vitro with 0 or 10 μM IAA for 3 h (n = 3 pools of 6 internodes 4–5 from 6 plants. Asterisks denote significant differences between IAA-treated and control-treated stems based on a post-hoc Kruskal–Wallis test (P < 0.001); (x 2) above IAA-treated stems refers to 2-fold change in comparison to control-treated stems. Different letters indicate significantly different results between control-treated genotypes based on a post-hoc Kruskal–Wallis test (P < 0.05). Data represent means ± SE. (D) Phylogenic tree of D27 proteins from rice (Os), Arabidopsis (At), pea (Ps), Vitis vinifera (Vv) and Physcomitrella patens. Protein sequences were aligned and used to generate the Maximum Likelihood phylogenetic tree with 1,000 bootstrap replicates. The percentage of replicate trees in which the associated taxa clustered together in the bootstrap test are shown next to the branches. Analyses were conducted in MEGA7. (TIF) [file pgen.1007089.s002.tif]

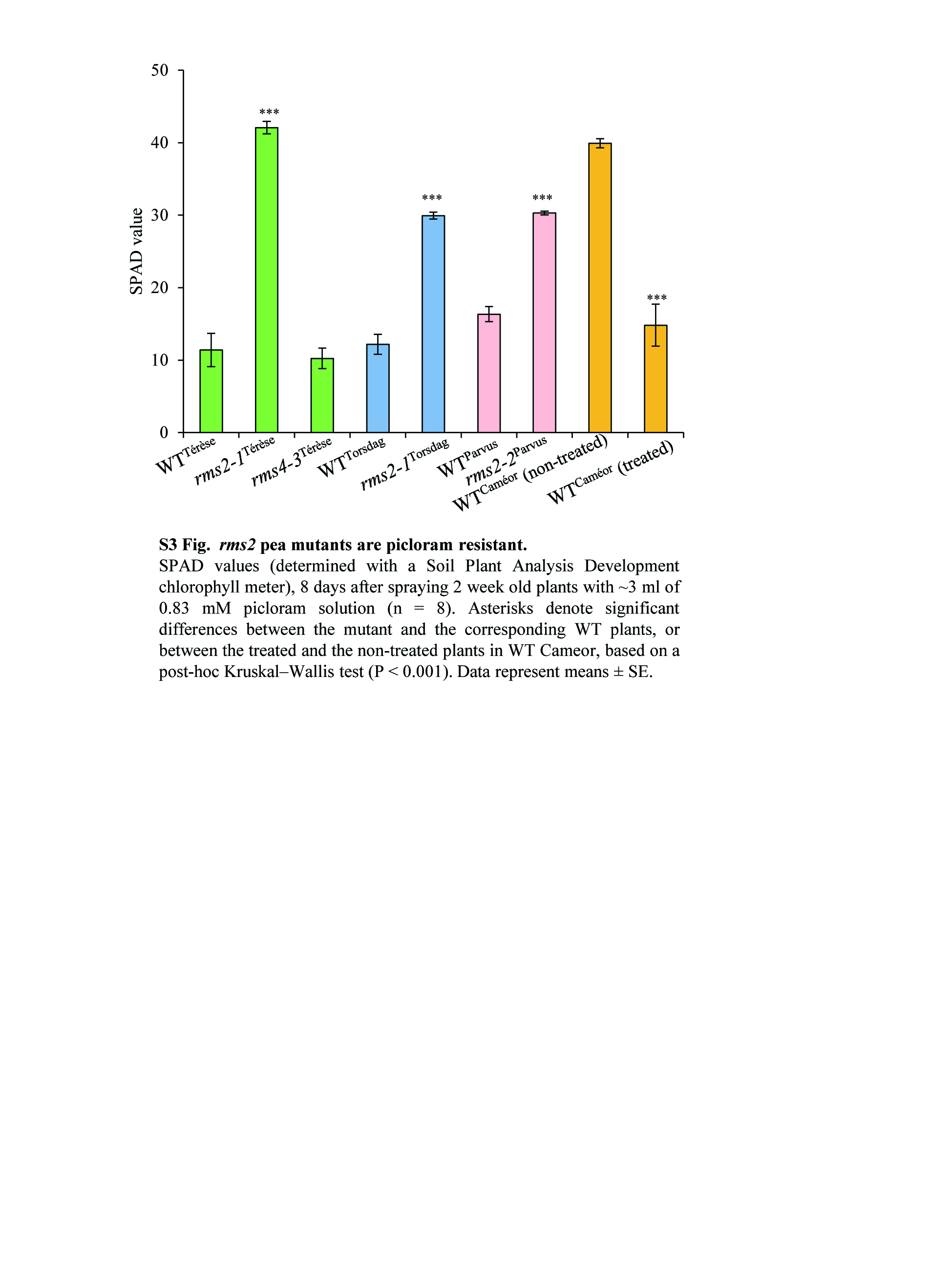

Supplement: S3 Fig — SPAD values (determined with a Soil Plant Analysis Development chlorophyll meter), 8 days after spraying 2 week old plants with ~3 ml of 0.83 mM picloram solution (n = 8). Asterisks denote significant differences between the mutant and the corresponding WT plants, or between the treated and the non-treated plants in WT Cameor, based on a post-hoc Kruskal–Wallis test (P < 0.001). Data represent means ± SE. (TIF) [file pgen.1007089.s003.tif]

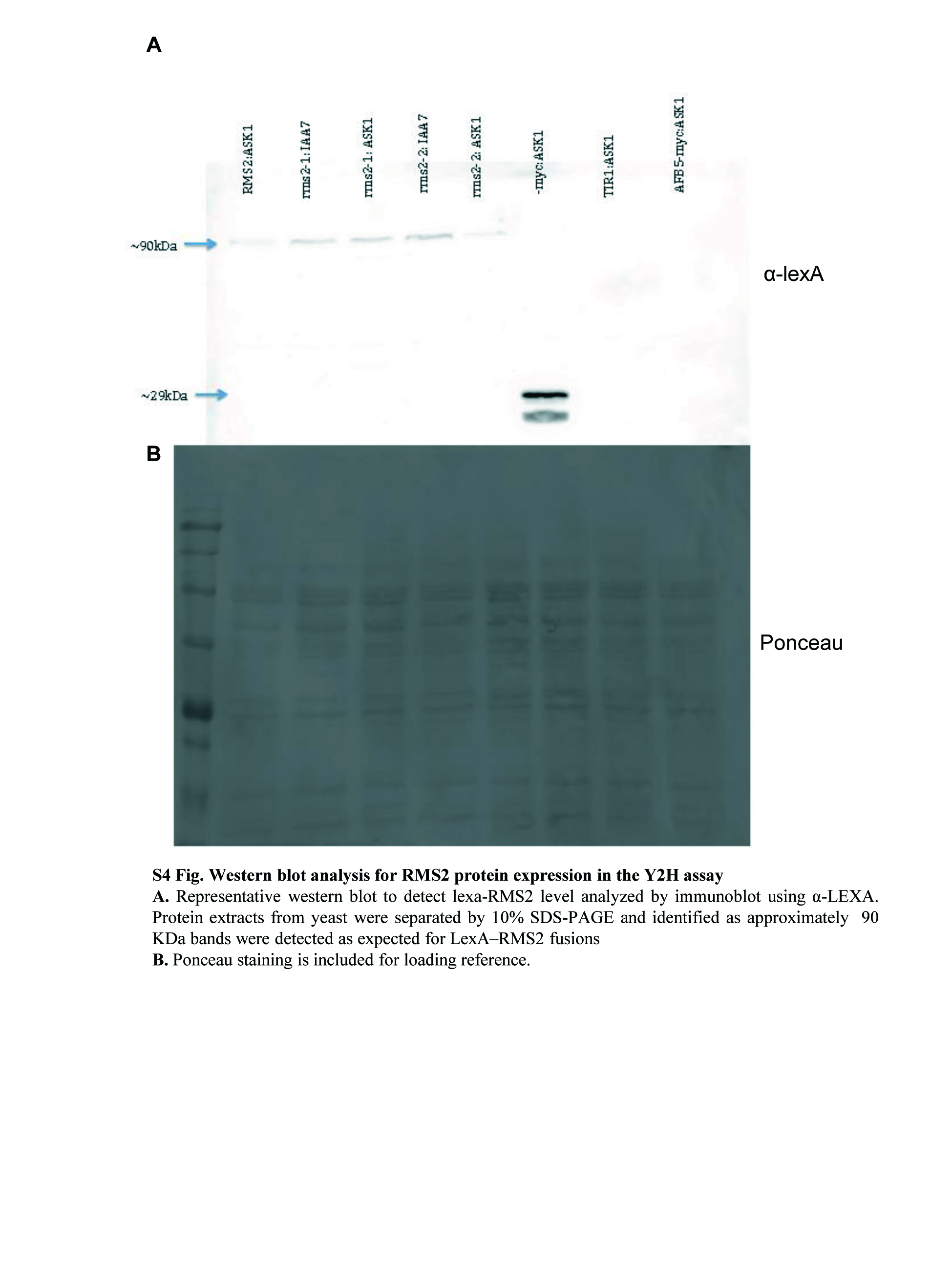

Supplement: S4 Fig — (A) Representative western blot to detect lexa-RMS2 level analyzed by immunoblot using α-LEXA. Protein extracts from yeast were separated by 10% SDS-PAGE and identified as approximately 90 KDa bands were detected as expected for LexA–RMS2 fusions. (B) Ponceau staining is included for loading reference. (TIF) [file pgen.1007089.s004.tif]

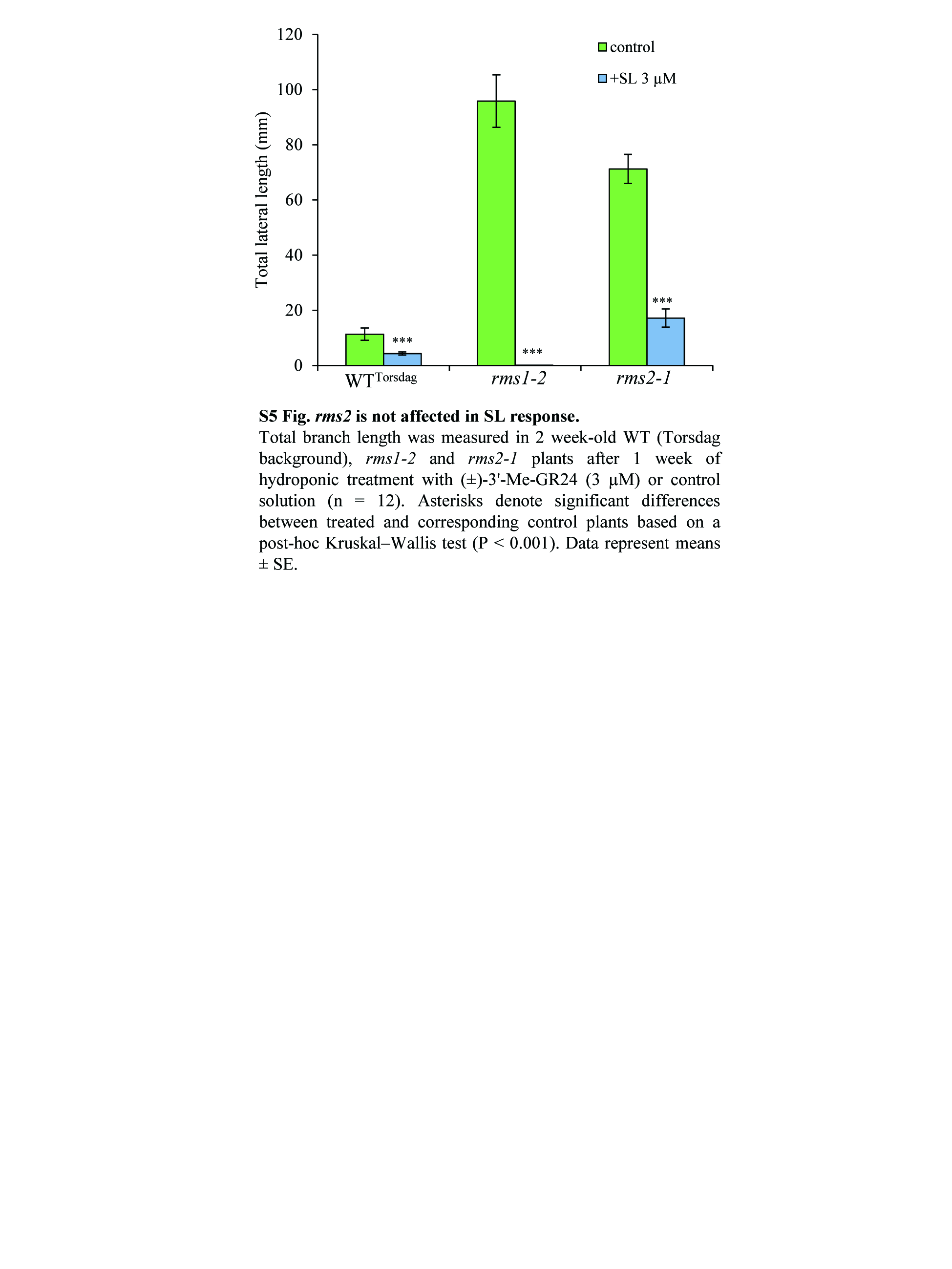

Supplement: S5 Fig — Total branch length was measured in 2 week-old WT (Torsdag background), rms1-2 and rms2-1 plants after 1 week of hydroponic treatment with (±)-3'-Me-GR24 (3 μM) or control solution (n = 12). Asterisks denote significant differences between treated and corresponding control plants based on a post-hoc Kruskal–Wallis test (P < 0.001). Data represent means ± SE. (TIF) [file pgen.1007089.s005.tif]

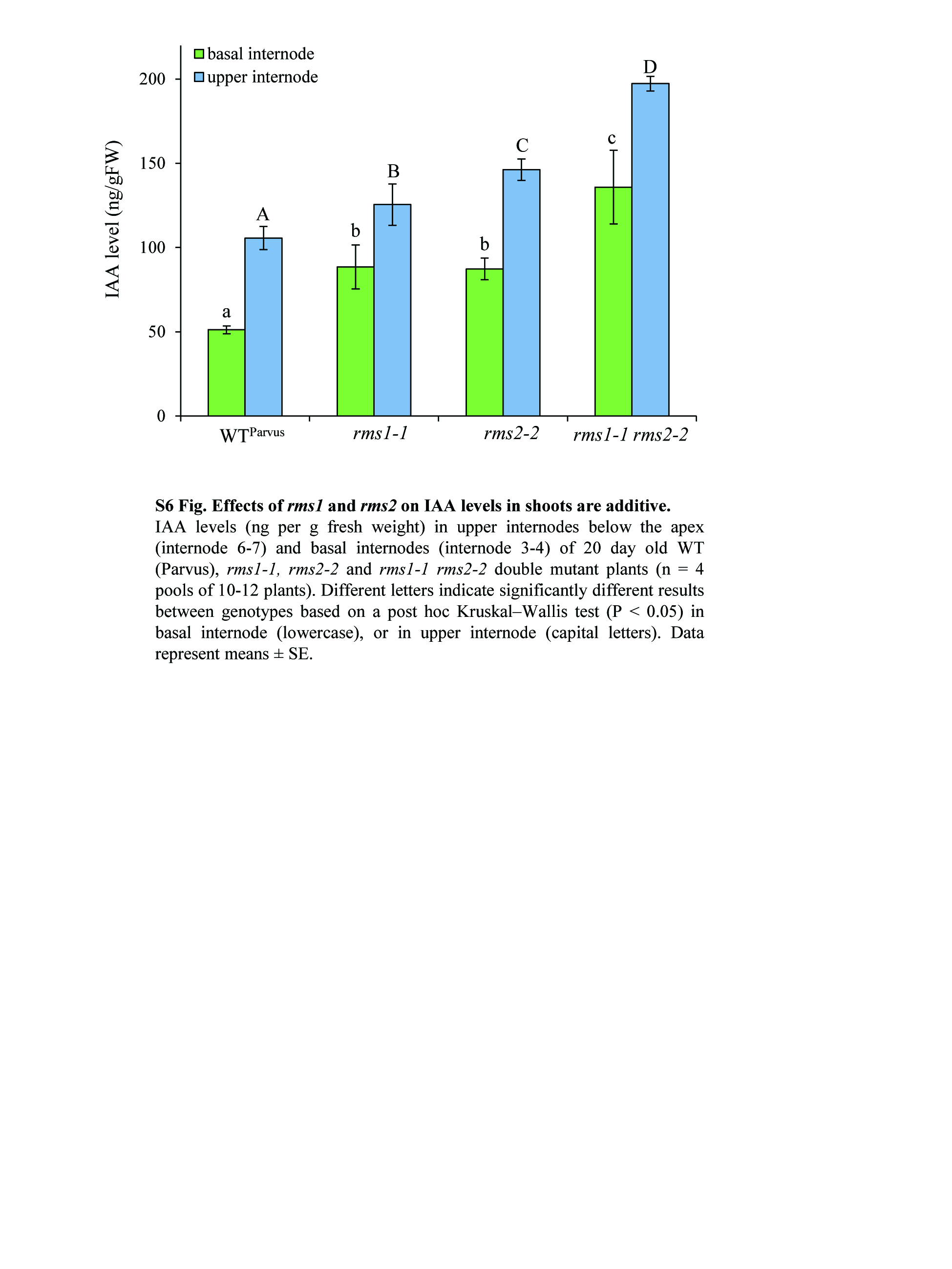

Supplement: S6 Fig — IAA levels (ng per g fresh weight) in upper internodes below the apex (internode 6–7) and basal internodes (internode 3–4) of 20 day old WT (Parvus), rms1-1, rms2-2 and rms1-1 rms2-2 double mutant plants (n = 4 pools of 10–12 plants). Different letters indicate significantly different results between genotypes based on a post hoc Kruskal–Wallis test (P < 0.05) in basal internode (lowercase), or in upper internode (capital letters). Data represent means ± SE. (TIF) [file pgen.1007089.s006.tif]

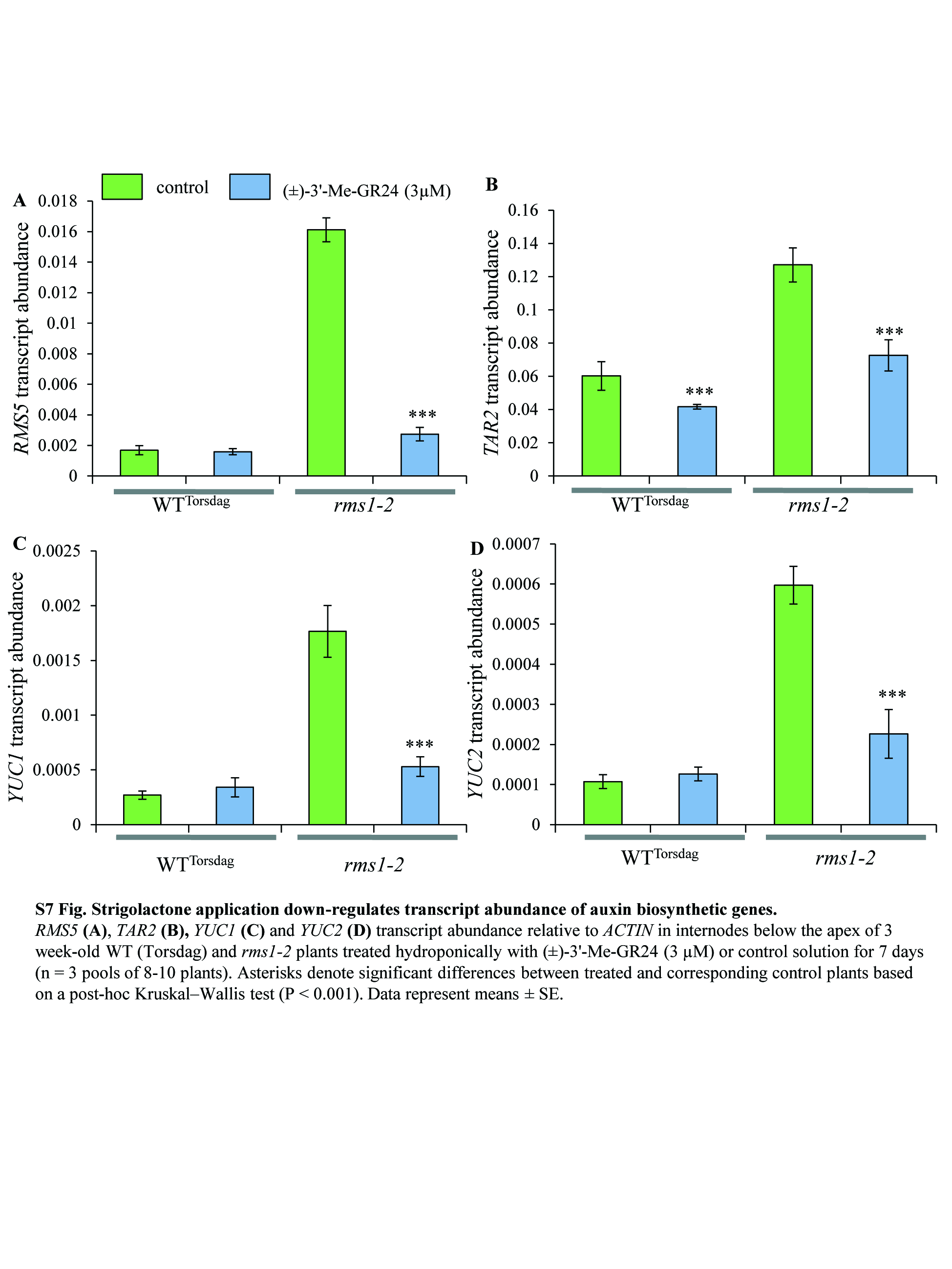

Supplement: S7 Fig — RMS5 (A), TAR2 (B), YUC1 (C) and YUC2 (D) transcript abundance relative to ACTIN in internodes below the apex of 3 week-old WT (Torsdag) and rms1-2 plants treated hydroponically with (±)-3'-Me-GR24 (3 μM) or control solution for 7 days (n = 3 pools of 8–10 plants). Asterisks denote significant differences between treated and corresponding control plants based on a post-hoc Kruskal–Wallis test (P < 0.001). Data represent means ± SE. (TIF) [file pgen.1007089.s007.tif]

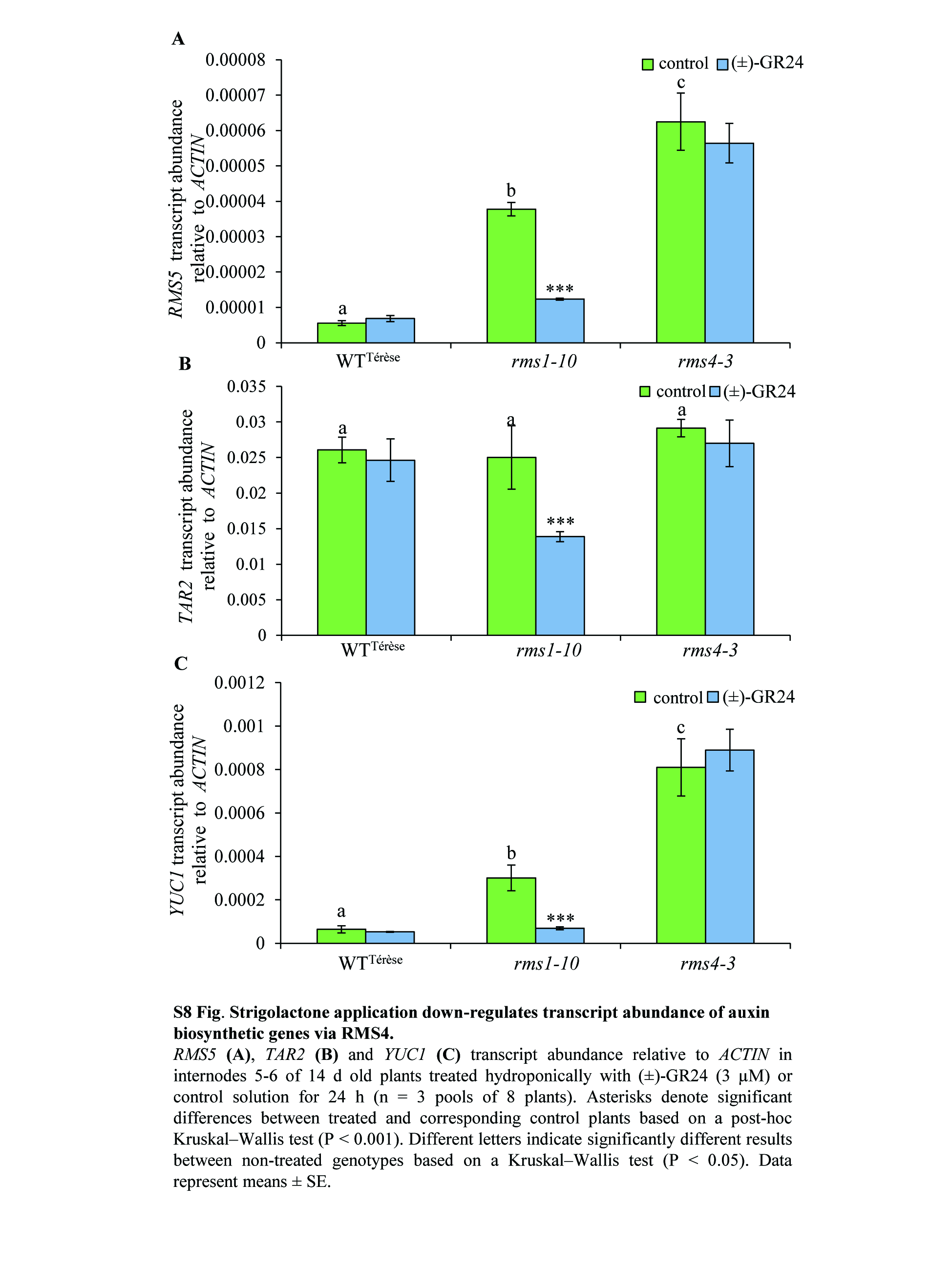

Supplement: S8 Fig — RMS5 (A), TAR2 (B) and YUC1 (C) transcript abundance relative to ACTIN in internodes 5–6 of 14 d old plants treated hydroponically with (±)-GR24 (3 μM) or control solution for 24 h (n = 3 pools of 8 plants). Asterisks denote significant differences between treated and corresponding control plants based on a post-hoc Kruskal–Wallis test (P < 0.001). Different letters indicate significantly different results between non-treated genotypes based on a Kruskal–Wallis test (P < 0.05). Data represent means ± SE. (TIF) [file pgen.1007089.s008.tif]
